# Gut microbiota dysbiosis shapes brain T-cell immunity in accelerated aging

**DOI:** 10.1080/19490976.2026.2728329

**Published:** 2026-09-17

**Authors:** Laura Peschke, Tinh Thi Nguyen, Jefferson Antônio Leite, Gwen Falony, Vu Thu Thuy Nguyen, Yogita Kattimani, Nishada Ramphal, Rebecca Jasser, Natalia Notarberardino Bos, Tommy Regen, Oliver Tüscher, Ari Waisman, Sara Vieira-Silva, Kristina Endres

**Affiliations:** a Institute of Medical Microbiology and Hygiene and Research Center for Immunotherapy (FZI), University Medical Center of the Johannes Gutenberg-University Mainz, Mainz, Germany; b Institute for Quantitative and Computational Biosciences (IQCB), Johannes Gutenberg-University Mainz, Mainz, Germany; c Institute of Molecular Biology, Mainz, Germany; d Department of Psychiatry and Psychotherapy, University Medical Center of the Johannes Gutenberg-University Mainz, Mainz, Germany; e Faculty of Computer Sciences and Microsystems Technology, Kaiserslautern University of Applied Sciences, Kaiserslautern, Germany; f Institute for Molecular Medicine, University Medical Center of the Johannes Gutenberg-University Mainz, Mainz, Germany; g Department of Immunology, Institute of Microbiology Paulo de Góes, Center of Health Sciences, Federal University of Rio de Janeiro (UFRJ), Rio de Janeiro, Brazil; h Host Microbe Interactomics Group, Wageningen University & Research, Wageningen, The Netherlands; i Leibniz Institute for Resilience Research, Mainz, Germany; j The University Clinic and Polyclinic for Psychiatry, Psychotherapy and Psychosomatics, University Medical Center Halle, Halle, Germany; k Systems Biology and Multiomics Research Group, Institute of Experimental and Clinical Research (IREC), UCLouvain, Brussels, Belgium

**Keywords:** Gut microbiota, dysbiosis, T-cell, aging, gut–brain axis

## Abstract

The bidirectional communication between the gut microbiota, immune system, and central nervous system—the gut‒brain axis—plays a vital role in maintaining brain health. Its disruption can lead to neuroinflammation and cognitive decline. However, the relationship between the gut microbiota and age-related changes in brain immunity remains unclear. This study examined gut microbiota composition and T-cell profiles in senescence-accelerated SAMP8 mice and senescence-resistant SAMR1 controls at young adult and aged stages. The accelerated-aging phenotype of SAMP8 mice was confirmed by reduced behavioral performance and brain transcriptomic alterations. Fecal microbiota profiles were obtained using culture-dependent plating and 16S rRNA gene sequencing. T-cell phenotypes were determined by flow cytometry on brain and splenic leukocytes. Strain, age, and sex were drivers of microbiota composition variation. Microbial diversity differed between strains independently of age. Several bacterial genera had altered abundances in young SAMP8 mice, with only *Intestinimonas* remaining higher in aged SAMP8. T-cell profiling revealed strain- and age-specific immune signatures. In the brain, SAMP8 mice exhibited a higher CD4⁺/CD8⁺ ratio, and higher levels of pro-inflammatory cytokine-producing CD4⁺ T cells compared to SAMR1. In contrast, the spleens of SAMP8 mice had a lower CD4⁺/CD8⁺ ratio. Only the spleens of aged SAMP8 mice showed increased T-cell activation (effector/naïve ratios) and higher levels of pro-inflammatory cytokine-producing CD4⁺ and CD8⁺ T cells, suggesting age- and tissue-specific immune alterations. Nine bacterial genera correlated with immune cell frequencies and ratios. Notably, *Intestinimonas* showed a discordant association with the CD4⁺/CD8⁺ ratio in the brain (negative) and spleen (positive). Furthermore, CD8⁺ T cells correlated with microbiota composition variation more strongly than strain, age, and sex. Together, these findings link the gut microbiota to age-dependent immune remodeling in the brain and imply that altered microbial communities could influence pro-inflammatory T-cell responses, thereby contributing to accelerated brain aging.

## 1. Introduction

The gut microbiota comprises a highly complex and dynamic community of microorganisms that profoundly influences physiological processes of the host.^1^ Beyond its well-established roles in digestion, the gut microbiota is recognized as a central regulator of neurological processes, via the gut–brain axis, a bidirectional communication network integrating microbial signals with neural, endocrine, and immune pathways that influence brain development, function, and behavior.^2^ ^,^ ^3^ One of the key routes of the gut–brain axis is immune modulation, notably through gut microbial metabolism of beneficial metabolites, such as short-chain fatty acids (SCFAs) or tryptophan derivatives, some of which contribute to the maintenance of intestinal barrier integrity and homeostasis of mucosal immunity.^3-5^

Alterations in the composition and function of gut microbial communities with impact on the host's health, termed dysbiosis, can disrupt these regulatory mechanisms, impacting systemic and central nervous system (CNS) inflammation.^6^ ^,^ ^7^ Dysbiosis has been associated with changes in microbial metabolism and altered release of microbial components that can modulate innate and adaptive immune pathways or even exert neurotoxic effects, thereby promoting neuroinflammatory processes.^8^ Neuroinflammation is a critical pathophysiological feature in aging-related cognitive decline and neurodegenerative disorders, including Alzheimer's and Parkinson's diseases.^9^ ^,^ ^10^ Experimental models have shown that dysbiosis can trigger glial activation, upregulate inflammatory signaling in the hippocampus and cortex, and impair synaptic function, which correlates with cognitive deficits.^11-13^

T cells function as a dynamic immunological relay between mucosal surfaces (especially within the gut) and the CNS under both homeostatic and inflammatory conditions. CD8⁺ cytotoxic T cells eliminate infected or damaged cells, whereas CD4⁺ helper T (Th) cells orchestrate immune responses by producing cytokines that shape innate and adaptive immunity. Within the CD4⁺ compartment, distinct cellular subsets (such as Th1, Th17, and regulatory T cells (Tregs)) exert pro-inflammatory or immunosuppressive functions, thereby balancing pathogen defense and immune tolerance.^14^ Within the gut–brain axis, dysregulated T-cell responses promote neuroinflammation as infiltrating effector T cells and their cytokines enhance microglial activation and contribute to neuronal dysfunction or injury.^15^ ^,^ ^16^ Aging further reshapes the T-cell compartment through immunosenescence, characterized by reduced naïve T-cell diversity, expansion of differentiated or senescent populations, and impaired regulatory capacity.^17^ These age-associated alterations contribute to chronic low-grade inflammation and have been linked to the pathogenesis of neurodegenerative diseases.^18^ The gut microbiota and its metabolic output are major environmental factors shaping T-cell activation and differentiation.^19^ Commensal microorganisms and their metabolites influence the development of effector and Treg subsets through antigen presentation and modulation of dendritic cell function. Specific bacteria promote Th17 differentiation,^20^ ^,^ ^21^ whereas others, e.g., *Clostridium*, induce Foxp3⁺ Tregs,^22^ contributing to immune homeostasis at mucosal and systemic levels. Dysbiosis can disrupt this tightly regulated crosstalk, leading to skewed T-cell polarization, impaired Treg induction, and enhanced effector responses, thereby promoting systemic inflammation.^23^

Despite extensive evidence linking gut dysbiosis, immune dysfunction, and neurodegeneration, the mechanistic interplay between dysbiosis-driven immune changes—especially those involving T-cell biology—and brain health remains poorly understood. A recent experimental study shows that intestinal inflammation promotes the expansion of microbiota-specific CD4⁺ T cells that infiltrate the brain and drive neuroinflammation.^24^ Once in the CNS, these cells produce pro-inflammatory cytokines, including granulocyte-macrophage colony-stimulating factor (GM-CSF), interferon gamma (IFNγ), and interleukin-17A (IL-17A), triggering microglial activation and neurological damage. Such findings suggest a potential link between gut-derived T-cell dysregulation and neuroinflammatory pathology, underscoring the need to clarify how microbiota-driven T-cell alterations contribute to cognitive decline and neurodegeneration. Addressing this gap is essential for deciphering the immunological pathways connecting gut microbiota to neurodegenerative diseases and for identifying potential therapeutic targets to preserve brain health during aging.

In the present study, we aim to identify gut microbiota features associated with aging-related changes in T-cell profiles in the brain. To this end, we used the senescence-accelerated mouse prone 8 (SAMP8) strain, a widely used model of accelerated aging, which exhibits early-onset cognitive decline, oxidative stress, and systemic inflammation,^25-28^ together with its control strain SAMR1 (senescence-accelerated mouse resistant 1). Gut microbiota composition was analyzed using complementary culture-dependent techniques and 16S rRNA gene sequencing, while T-cell populations in the brain and the spleen were assessed by flow cytometry. Our findings demonstrate a strong link between gut microbiota composition and immune dynamics in the brain during aging, thereby broadening our understanding of microbiota–immune interactions in the context of brain aging.

## 2. Materials and methods

### 2.1. Animals and study design

SAMP8 (RRID:IMSR_ENV:HSD-954) and control SAMR1 (RRID:IMSR_ENV:HSD-956), male and female mice, originating from Envigo (Indianapolis, IN, USA), were bred under a license in the facility of Johannes Gutenberg University (TARC). Mice were maintained on a 12-hour light/dark cycle and were provided with food (ssniff Spezialdiäten, Soest, Germany) and water *ad libitum*. Animals were kept in groups of maximally five per cage.

This study included four independent cohorts of SAMP8 and SAMR1 mice. The first cohort comprised a longitudinal cohort of female and male SAMP8 and SAMR1 mice that was investigated at 3, 5, 7, and 10 months of age for repeated assessment of grip strength and culture-based quantification of viable fecal bacteria. In addition, a smaller cohort of female and male SAMP8 and SAMR1 mice at 10 months of age was used to assess T-maze performance. Based on the age-dependent characterization of viable fecal bacterial populations, representative young adult (14–19 weeks) and aged (33–52 weeks) stages were selected for subsequent comprehensive microbiome and immune profiling in an independent cross-sectional cohort. This third cohort included female and male SAMP8 and SAMR1 mice and was used for fecal 16S rRNA gene sequencing to characterize gut microbiota composition and predicted functional profiles, as well as tissue-specific T-cell characterization in brain and spleen. A fourth independent cross-sectional cohort was used to further validate molecular signatures associated with accelerated aging and included male SAMP8 and SAMR1 mice at 3 and 10 months of age for brain transcriptomic profiling.

No *a priori* power analysis for sample size was performed. Sample sizes were determined based on experiment objectives. The longitudinal cohort was established to characterize the newly established SAMP8 and SAMR1 colonies in our animal facility by monitoring health status, behavioral performance, and gut microbiota over the course of aging. Sample sizes for the cross-sectional experiments were selected because these analyses were exploratory and performed for the first time in this mouse model, with the aim of generating preliminary evidence for future hypothesis-driven studies, while minimizing animal use in accordance with the principles of the 3Rs. During the longitudinal study, mice that died before completion of the experiment were excluded from subsequent analyses. No other predefined inclusion or exclusion criteria were applied. The study groups were not blinded during experimental procedures or data collection. The individual mouse was considered the experimental unit for all analyses. The number of animals included in each analysis is specified in the respective experimental sections, figure legends, and supplementary tables.

All procedures were performed by trained personnel in accordance with the European Communities Council Directive regarding care and use of animals, as well as institutional and national animal welfare guidelines, to minimize pain, suffering, and distress. Animals were monitored twice per week by staff using a standardized welfare assessment. Behavioral experiments were approved by the ethics committee of the State Investigation Office Rhineland-Palatinate (Landesuntersuchungsamt Rheinland-Pfalz) under protocol number E3 G-19-1-025. Animals used for tissue collection were euthanized for organ harvest in accordance with §4 of the German Animal Welfare Act. Animals were euthanized by CO₂ inhalation according to institutional guidelines for immune profiling experiments and euthanized by isoflurane inhalation followed by decapitation for transcriptomic analyses. Tissues were immediately harvested for downstream analyses as described below.

### 2.2. Behavioral characterization

Behavioral tests were conducted according to a fixed schedule. SAMP8 and SAMR1 mice at 3, 5, 7, and 10 months of age were used for the grip strength test (total *N* = 336; detailed group sizes are provided in Supplementary Table S01). SAMP8 and SAMR1 mice at 10 months of age were used for the T-maze test (*N* = 17; *n* = 8 [3 males, 5 females] for SAMR1; *n* = 9 [4 males, 5 females] for SAMP8). The grip strength and T-maze tests were performed using the apparatus and protocols as previously described in our studies.^29^ ^,^ ^30^

### 2.3. Brain transcriptomic characterization

Male SAMP8 and SAMR1 mice at 3 and 10 months of age (*n* = 4 per age and genotype; independent transcriptomic cohort) were euthanized by isoflurane inhalation and subsequently decapitated using a guillotine. One brain hemisphere was dissected, and the prefrontal cortex was isolated for transcriptomic profiling. Tissue samples were immediately preserved in RNAprotect Tissue Reagent (Qiagen, Cat #76104) and stored at −80 °C until processing. RNA was isolated using the RNeasy Mini kit (Qiagen, Cat #74104) and RNase-free DNase set (Qiagen, Cat #79254) treatment according to the manufacturer's instructions. Library prep was performed with Illumina Stranded mRNA Prep Kit following Illumina Stranded mRNA Prep Reference Guide (April 2021) (Document # 1000000124518 v02). Libraries were prepared with a starting amount of 1000 ng and amplified in 10 PCR cycles. Libraries were profiled in a DNA high-sensitivity chip on a 2100 Bioanalyzer (Agilent Technologies, Cat #G2939BA) and quantified using the Qubit 1x dsDNA HS Assay Kit (Invitrogen by Thermo Fisher Scientific), in a Qubit 4.0 fluorometer (Invitrogen by Thermo Fisher Scientific, Cat #Q33230). Libraries were sequenced on an Illumina NextSeq2000 P1 (Illumina, Cat #20050264) and demultiplexed using bclfastq (v.2.19). Sequencing reads were aligned to the *Mus musculus* reference genome (GRCm39) using STAR (v2.7.10b) with Ensembl release 109 gene annotation. Gene-level read counts were generated using featureCounts from the Subread package, assigning uniquely mapped reads overlapping annotated gene features (exons and introns) on the same strand. Differential gene expression analysis was performed using Bioconductor (v3.18)/DESeq2 (v.1.52.0). DESeq2's default independent filtering procedure was applied to remove lowly expressed genes and improve statistical power. RPKM (Reads per kilobase of transcript per million mapped reads) were calculated per gene based on FPM (robust counts per million mapped fragments) normalized to gene length. Differentially expressed genes were identified using a false discovery rate (FDR; Benjamini–Hochberg (BH) adjusted *P* value) threshold of <0.01, with the fold-change threshold set to 1, consistent with current recommendations to avoid post hoc fold-change filtering and minimize inflated FDRs.

### 2.4. Culture-based quantification of viable fecal bacteria

Viable bacterial abundance in the gastrointestinal tract was determined using fecal samples collected from SAMP8 and SAMR1 mice at 3, 5, 7, and 10 months of age (total *N* = 336; same cohort as used for grip strength; detailed group sizes are provided in Supplementary Table S02). Representative gut commensal families, including *Lactobacillaceae*, *Enterobacteriaceae*, and Schaedler flora, were analyzed according to established methods.^29^

### 2.5. Microbiome and T-cell profiling

The primary microbiome-immune profiling cohort consisted of male and female SAMP8 and SAMR1 mice at young adult (aged 14–19 weeks) and aged (aged 33–52 weeks) stages (*N* = 32; *n* = 4 per sex, per genotype, per age). Animals from this cohort were used for fecal 16S rRNA gene sequencing as well as tissue-specific T-cell characterization in brain and spleen.

#### 2.5.1. DNA isolation and sequencing of fecal materials

Feces were collected from the colon of the dissected mice and stored at −80 °C prior to DNA extraction. DNA was isolated from fecal samples using the QIAamp Fast DNA Stool Mini Kit (Qiagen, Cat #51604) following the manufacturer's instructions. Libraries were prepared with a starting amount of 15 ng whole genomic DNA. The V4 region of the 16S rRNA gene was amplified by PCR using the 515F/806R primer pair, containing part of Illumina Read1- & Read2-adaptors, respectively, as well as heterogeneity spacers to increase sequence diversity. Libraries were completed in a subsequent 15-cycle PCR, in which unique-dual index barcodes (UDIs) were added to the libraries. Libraries were profiled in a DNA high-sensitivity chip on a 2100 Bioanalyzer (Agilent Technologies, Cat #G2939BA) and quantified using the Qubit 1x dsDNA HS Assay Kit (Invitrogen by Thermo Fisher Scientific, Cat #Q33230), in a Qubit 4.0 fluorometer (Invitrogen by Thermo Fisher Scientific, Cat #Q33238). All 32 libraries were pooled together in equimolar ratio. The pool was purified with magnetic beads in a 1:1-ratio (sample:bead) to remove residual primers and adapter dimers and sequenced on an Illumina NextSeq2000 P1 (300 cycles) kit (Illumina, Cat #20050264), in 2 × 161 cycles for the paired-end reads plus 2 × 8 cycles for the unique dual index reads, obtaining around 1–2.5 million paired-end reads per sample.

#### 2.5.2. Microbiota taxonomic and functional profiling

Data processing and annotation were performed using the DADA2 pipeline (v.1.32.0, RRID:SCR_023519).^31^ Raw reads were initially pre-filtered to remove sequences containing ambiguous bases (maxN = 0), followed by primer removal using Cutadapt (RRID:SCR_011841)^32^ with a minimum overlap of 15 bp. Subsequent quality filtering was conducted in DADA2 using an expected error threshold of 2 for both forward and reverse reads (maxEE = c(2,2)) and a minimum length of 100 bp. Error rate learning, amplicon sequence variant (ASV) inference, paired-end merging, sequence table construction, and chimera removal were performed using default DADA2 parameters. Taxonomic assignment was performed using the Genome Taxonomy Database (GTDB, v.86). Functional profiles were predicted from ASVs using PICRUSt2 (v.2.6.3; Python v.3.12.13) to infer MetaCyc metabolic pathways, using the default PICRUSt2-SC database and default phylogenetic placement and pathways inference procedures.^33-38^

#### 2.5.3. Spleen cell isolation

Spleens were collected from euthanized mice and transferred to a 50 mL tube containing cold PBS. The tissues were gently mashed through a 70 μm cell strainer using a syringe plunger (Becton Dickinson, Cat #14-823-30) to obtain a single-cell suspension. Cells were then centrifuged at 450 × g for 5 minutes at 4 °C, and the red blood cells were lysed by incubating the pellet in 1 × ACK lysis buffer (ammonium–chloride–potassium buffer; 150 mM NH₄Cl, 10 mM KHCO₃, 0.1 mM EDTA, pH 7.2–7.4) for 3 minutes at room temperature. The reaction was stopped by adding 10 mL of cold PBS, followed by centrifugation at 450 × g for 5 minutes. The resulting splenocyte pellet was resuspended in PBS for downstream staining and flow cytometry analysis.

#### 2.5.4. Brain-infiltrating immune cell isolation

Brains including olfactory bulbs and cerebellum were harvested and dissected into 1–2 mm fragments in a Petri dish containing PBS. Tissue fragments were transferred to a 50 mL tube, briefly centrifuged, and resuspended in 1–2 mL of PBS containing 1 mg/mL collagenase D (Roche, Cat #11088858001) and 10–15 U/mL DNase I (Roche, Cat #10104159001). Samples were incubated for 20 minutes at 37 °C with gentle agitation. After enzymatic digestion, the tissue was homogenized by passing it through a 5 mL syringe (BD Biosciences, Cat #309646) fitted with an 18–20 G needle. The homogenate was diluted with PBS and centrifuged at 280 × g for 5 minutes at room temperature. The pellet was carefully resuspended in 4.5 mL of 70% isotonic Percoll (Cytiva, Cat #17-0891-01) and further homogenized. The resulting suspension was gently underlaid below 4 mL of 37% Percoll, which have been previously underlaid beneath 4 mL of 30% Percoll in a 15 mL conical tube, forming a three-layer discontinuous Percoll gradient. Samples were centrifuged at 500 × g for 30 minutes at room temperature with no brake. After centrifugation, the myelin layer and upper ring were carefully removed using a vacuum system. The red layer and the distinct white interphase ring (containing lymphocytes and microglia) were collected into a clean 15 mL tube, taking care to avoid disturbing the pellet at the bottom. This cell suspension was washed with PBS containing 0.5  mM EDTA (Thermo Fisher Scientific, Cat #15575-020), centrifuged again at 280 × g for 5 minutes, and resuspended in PBS. A final spin at 450 × g for 5 minutes was performed in a benchtop centrifuge, and the resulting pellet was used for flow cytometry. Our tissue preparation protocol did not include removal of the meninges; therefore, the isolated immune cells may include both parenchymal and meningeal-associated populations.

#### 2.5.5. Flow cytometry (FACS) analysis

Quantitative and phenotypic analysis of lymphocytes isolated from spleen and brain was performed by flow cytometry. After isolation, cells were counted and viability was assessed by trypan blue exclusion. A total of 2 × 10^6^ cells were plated in 96-well round-bottom plates and stimulated for 4 hours at 37 °C (5% CO₂) with phorbol-12-myristate-13-acetate (PMA, 50 ng/mL; Sigma-Aldrich, Cat #P8139), ionomycin (500 ng/mL; Sigma-Aldrich, Cat #I3909), and brefeldin A (1.5 μL/mL; BD Biosciences, Cat #555029) to promote intracellular cytokine accumulation. After stimulation, cells were washed twice with PBS, centrifuged at 450 × g for 5 minutes, and incubated with Fc block (anti-CD16/32, clone 93, dilution 1:200; BioLegend, Cat #101307) for 15 minutes at room temperature. Dead cells were excluded using a fixable viability dye (APC-eFluor 780, dilution 1:1000; eBioscience, Cat #65-0865). Surface staining was performed for 20 minutes at 4 °C in PBS using the following fluorochrome-conjugated antibodies: anti-CD45 BV510 (clone 30-F11, dilution 1:200; BioLegend, Cat #103137), anti-CD4 PE-Cy7 (clone GK1.5, dilution 1:200; BioLegend, Cat #100421), anti-CD4 BV421 (GK1.5, dilution 1:500; BioLegend, Cat #11602), anti-CD4 PE (clone RM4-5, dilution 1:400; BioLegend, Cat #100511), anti-CD62L FITC (clone MEL-14, dilution 1:200; BioLegend, Cat #104405), anti-CD44 PerCP-Cy5.5 (clone IM7, dilution 1:100; BioLegend, Cat #103031), and anti-CD3 PerCP (clone eBio17B7, 1:1000; eBioscience, Cat #46-0032-82). Cells were then fixed for 60 minutes at room temperature using the Fixation/Permeabilization buffer (eBioscience, Cat #00-5523-00), followed by centrifugation and resuspension in the permeabilization buffer provided in the same kit. Intracellular staining was performed for 60 minutes at 4 °C using antibodies: anti-Foxp3 APC (clone FJK-16s, dilution 1:400; Thermo Fisher Scientific, Cat #17-5773-82), anti-IL-17A (clone eBio17B7, dilution 1:400; eBioscience, Cat #48-7177-82), and anti-IFN-*γ* FITC (clone XMG1.2, dilution 1:400; BioLegend, Cat #505805). After washing once with PBS, cells were resuspended in PBS and analyzed on a FACSCanto II flow cytometer (BD Biosciences; RRID:SCR_018056). Between 100,000 and 1,000,000 events were acquired per sample. Data were analyzed using FlowJo X software (BD Biosciences).

### 2.6. Statistical analyses

Behavioral tests and culture-based analyses of fecal microbiota composition were performed using GraphPad Prism version 10 software (https://www.graphpad.com/; RRID:SCR_002798) for Windows. Outliers were identified and removed using the ROUT method with an FDR of 5%. Data were assessed for normality using the D'Agostino-Pearson omnibus normality test. When the assumption of normality was not met, non-parametric tests were used as appropriate, including the unpaired two-tailed Mann–Whitney U test for T-maze comparisons and the Kruskal–Wallis test followed by Dunn's multiple comparisons test for culture-based analyses of fecal microbiota composition. When the assumption of normality was met, homogeneity of variances was assessed using the Brown-Forsythe test. Welch's ANOVA was used when the assumption of equal variances was violated, as was the case for the grip strength analysis. Statistical significance findings were set for the comparison between the genotypes as follows: *, *P* < 0.05; **, *P* < 0.01; and ***, *P* < 0.001.

Statistical analyses for microbiota data derived from 16S rRNA gene sequencing and T-cell data were performed in R (v.4.5.2; RRID:SCR_001905) using the following packages: *vegan* v.2.7.1 (RRID:SCR_011950), *phyloseq* v.1.52.0 (RRID:SCR_013080), and *FSA* v.0.10.1 (RRID:SCR_016114). Unless otherwise specified, all analyses on the primary microbiome-immune profiling cohort were performed using data from *N* = 32 mice (*n* = 4 per sex, per genotype, per age). Analyses involving T-cell data were performed using *N* = 31 mice (*n* = 8 young SAMR1, *n* = 8 aged SAMR1, *n* = 8 young SAMP8, *n* = 7 aged SAMP8) as one sample was removed during FACS analysis. Because microbiota and immune cell data were not assumed to follow a normal distribution, nonparametric statistical methods were applied in all analyses. Differences between study groups were assessed using Kruskal–Wallis tests (reporting ordinal epsilon squared (ε^2^_R_), calculated from the test-derived H-statistic divided by sample size minus one) with post hoc Dunn's tests conducted to assess pairwise differences between study groups (reporting standardized effect size r, calculated as the test-derived Z-statistic divided by the square root of sample size). Spearman's rank correlation tests were used to test rank-order correlations between continuous variables (reporting correlation coefficient rho). All tests were two-sided. *P* values from the Kruskal‒Wallis and Spearman's tests were corrected for multiple testing using the BH method (*P*_adj_). Pairwise Dunn's post hoc tests were evaluated using unadjusted *P* values. Only *P*_adj_ < 0.05 for Kruskal–Wallis and Spearman's tests and *P* < 0.05 for post hoc Dunn's tests were reported as significant. To identify candidate genera and pathways for follow-up analyses, Kruskal‒Wallis results with *P*_adj_ < 0.1 were considered suggestive and are explicitly reported as such in the results. Leave-one-out (LOO) sensitivity analyses were performed for all significant results by retesting iteratively after removing one sample at a time.

All visualizations were carried out using the R packages *ggplot2* v.4.0.1 (RRID:SCR_014601) and *phyloseq* v.1.52.0.

#### 2.6.1. Partitioning of microbiota composition variation across explanatory variables

The estimation of the explanatory power of metadata variables regarding relative, genus-level microbiota composition variation was performed using single or multivariable stepwise distance-based redundancy analysis (dbRDA; Bray‒Curtis dissimilarity) as implemented in the R package *vegan*. Interindividual microbiota composition variation was visualized by principal coordinates analysis (PCoA) using Bray-Curtis dissimilarity on the genus-level relative abundance matrix (R package *phyloseq*).

#### 2.6.2. Microbiota features and metadata associations

Microbial diversity was estimated at the genus level as the Shannon index (R package *phyloseq*). For analyses of genus abundances, taxa unclassified at the genus level or present in fewer than 50% of samples within each study group were excluded from statistical testing. For analyses of pathway abundances, pathways present in fewer than 80% of samples within each study group were excluded from statistical testing.

#### 2.6.3. Multivariable analysis of T-cell composition in the brain and spleen

To identify covariates of tissue-specific variation in T-cell composition and to estimate the explanatory power of metadata variables, single or multivariable stepwise redundancy analysis (RDA) was performed using tissue-specific T-cell frequencies (scaled). Interindividual variation of T-cell composition was visualized by principal component analysis (PCA).

## 3. Results

**Figure 1.**
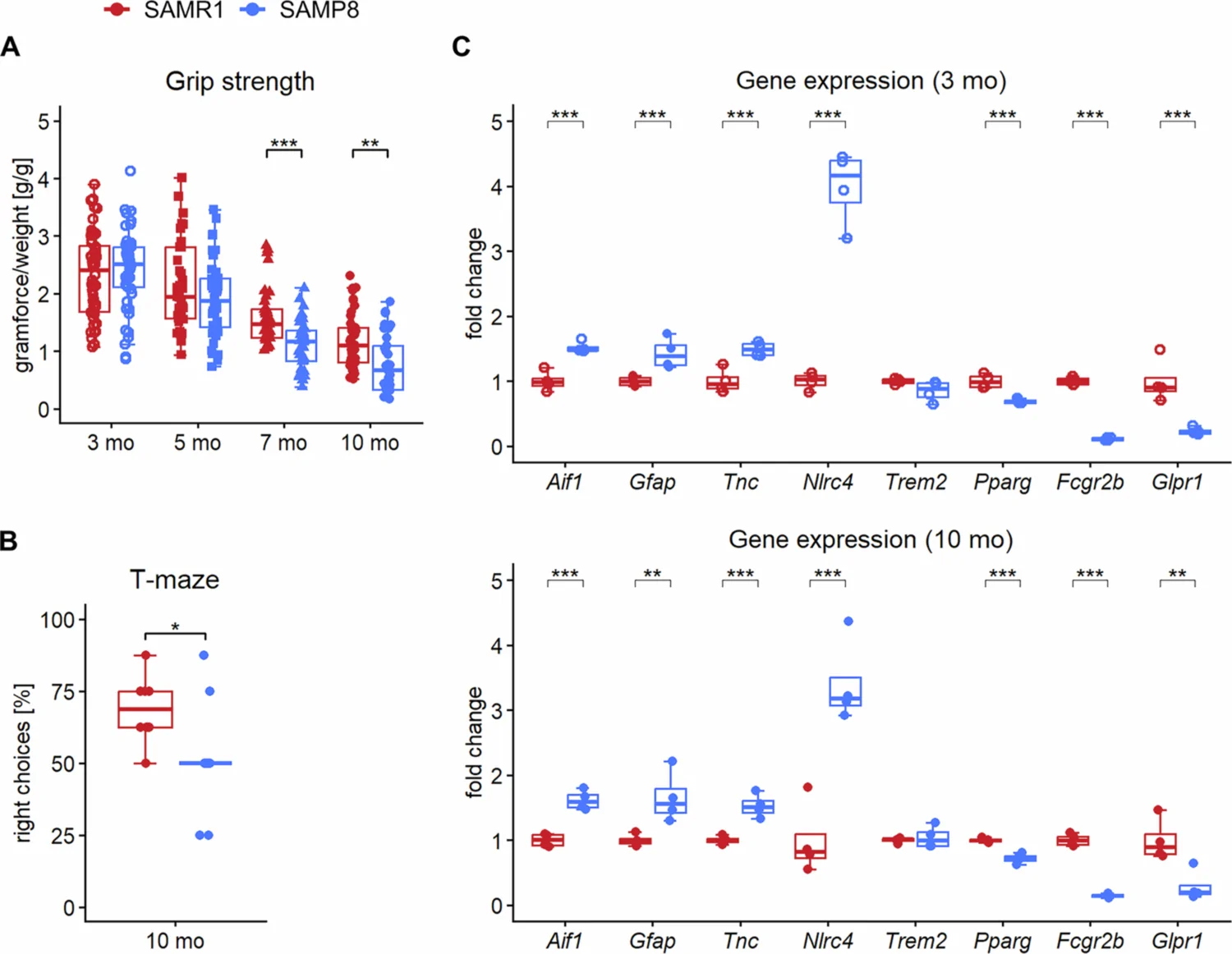
Phenotypic characterization of accelerated aging in SAMP8 mice compared with SAMR1 mice. A, Grip strength of SAMP8 and age-matched SAMR1 mice at 3, 5, 7, and 10 months of age. Grip strength was measured using a Newton meter and normalized to body weight (gramforce/weight). Data are presented as mean ± SEM. Statistical analyses were performed using Brown–Forsythe and Welch ANOVA followed by Dunnett's T3 multiple-comparisons test (*N* = 336; *n* = 36–52, male and female for SAMR1 and SAMP8). B, Spatial working memory assessed by the T-maze test in 10-month-old SAMP8 and SAMR1 mice. Data are presented as the percentage of right choice (mean ± SEM). Statistical analyses were performed using an unpaired two-tailed Mann‒Whitney U test (*N* = 17; *n* = 8 [3 males, 5 females] for SAMR1; *n* = 9 [4 males, 5 females] for SAMP8). C, Representative transcriptomic analysis of prefrontal cortex genes in male 3- and 10-month-old SAMP8 mice, presented as fold change relative to age-matched SAMR1 mice (*n* = 4 animals per age, genotype). Differentially expressed genes were identified using the DESeq2 package and a BH-adjusted *P* value < 0.01. Exact *P* values and sample sizes are provided in Supplementary Table S01. *, *P* < 0.05; **, *P* < 0.01; ***, *P* < 0.001. Abbreviation: mo, months.

Using SAMP8 mice as a model of accelerated aging and SAMR1 mice as normal-aging controls, we examined gut microbial dynamics and tissue-specific T-cell composition in young and old adults to define immunological and microbial signatures of the aging trajectory. To reconfirm the accelerated-aging phenotype of the SAMP8 model, we assessed behavioral performance, including grip strength as an indicator of age-related neuromuscular function (Figure 1A) and the T-maze test as a measure of spatial working memory (Figure 1B), together with an exemplary transcriptomic analysis of brain gene-expression markers associated with glial activation, neuroinflammation, and brain homeostasis (Figure 1C), in comparison with age-matched SAMR1 mice. Grip strength was measured using a Newton meter and expressed as gram force normalized to body weight. Grip strength was comparable between SAMP8 and SAMR1 mice at younger ages (3 and 5 months) but was significantly reduced in SAMP8 mice at 7 months (*P*_adj_ = 0.0001; Figure 1A; Supplementary Table S01) and 10 months of age (*P*_adj_ = 0.0013; Figure 1A; Supplementary Table S01). Similarly, SAMP8 mice exhibited impaired spatial working memory in the T-maze test. Whereas SAMR1 mice correctly selected the alternating arm in 68% of trials, SAMP8 mice achieved only approximately 51% right choices (*P*_adj_ = 0.0104; Figure 1B; Supplementary Table S01). These findings confirm the progressive decline in neuromuscular performance and cognitive function characteristic of accelerated aging in SAMP8 mice.^11^ ^,^ ^27^ ^,^ ^28^ ^,^ ^39^ ^,^ ^40^ To further characterize brain aging, we analyzed the expression of representative genes associated with glial activation, neuroinflammation, and immune homeostasis (Figure 1C; Supplementary Table S01). These included *Aif1* (Allograft inflammatory factor 1) as a marker of microglial activation^41^; *Trem2* (Triggering receptor expressed on myeloid cells 2 ), a microglia-enriched receptor involved in microglial activation and disease-associated microglial responses^42^; *Gfap* (Glial fibrillary acidic protein) as a marker of astrocyte activation^43^; *Tnc* (Tenascin C) and *Nlrc4* (NLR family CARD domain containing 4) as inflammation-associated genes^44^ ^,^ ^45^; and *Pparg* (Peroxisome proliferator-activated receptor gamma),^46^ *Fcgr2b* (Fc gamma receptor IIb),^47^ and *Glp1r* (Glucagon-like peptide 1 receptor)^48^ as genes involved in immune regulation and neuroprotective signaling. Compared with SAMR1 mice at 3 and 10 months of age, SAMP8 mice exhibited significantly increased expression of *Aif1*, *Gfap*, *Tnc*, and *Nlrc4*, unchanged expression of *Trem2*, and decreased expression of *Pparg*, *Fcgr2b*, and *Glp1r* (Figure 1C; Supplementary Table S01). Collectively, these behavioral and transcriptomic findings further validate the SAMP8 mouse as a model of accelerated aging.

### 3.1. Gut microbiota dysbiosis in accelerated aging

**Figure 2.**
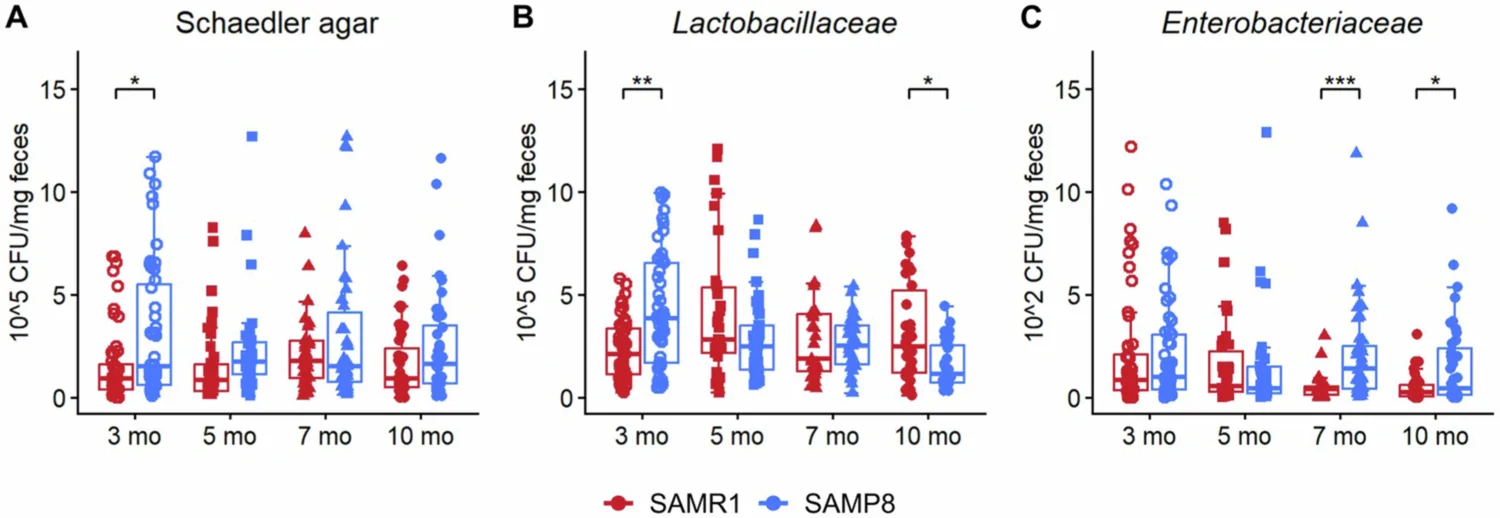
Culture-based view of fecal microbiota composition in SAMR1 and SAMP8 mice at different ages. Fecal pellets were collected at 3, 5, 7, and 10 months of age (*N* = 335; *n* = 27–52, male and female for SAMR1 and SAMP8), plated on selective media, and quantified as colony-forming units (CFUs) per mg of feces. CFUs grown on selective agar for *Lactobacillaceae* (A), on Schaedler agar (B), and on agar selective for *Enterobacteriaceae* (C). Statistical analysis was performed by Kruskal–Wallis test followed by Dunn's multiple comparisons test (Supplementary Table S02); *, *P* < 0.05; **, *P* < 0.01; ***, *P* < 0.001). Abbreviations: mo, months.

To obtain an initial view of gut microbiota changes associated with chronological age and age-related functional decline, we first carried out culture-based analyses of bacterial groups across the lifespan. Total bacteria (cultured on the broad anaerobic cultivation medium Schaedler agar), *Lactobacillaceae* (generally beneficial gram-positive, lactic acid-producing bacteria), and *Enterobacteriaceae* (generally gram-negative facultative anaerobes, with some opportunistic pathogens) were quantified as colony-forming units (CFUs) to estimate the viable bacterial load in fecal samples. Fecal samples from SAMP8 mice were compared with those from age-matched SAMR1 controls at 3, 5, 7, and 10 months of age to identify age-dependent differences of viable bacterial populations and thereby guide the selection of age groups for subsequent microbiome amplicon profiling. Overall, culture-based analyses suggested that the largest differences between SAMP8 and SAMR1 occurred early in life. At 3 months, SAMP8 mice exhibited higher counts of anaerobic gut commensals cultured on Schaedler agar (*P*_adj_ = 0.0405; Figure 2A; Supplementary Table S02) and increased *Lactobacillaceae* (*P*_adj_ = 0.0021; Figure 2B) compared with SAMR1 mice. In contrast, total Schaedler counts were comparable between mouse strains at 5, 7 and 10 months, indicating that the overall cultivable microbiota became more similar with age. Nevertheless, at 7 months of age, SAMP8 mice showed significantly higher counts of the *Enterobacteriaceae* family (*P*_adj_ = 0.0007; Figure 2C). At 10 months of age, SAMP8 mice displayed higher *Enterobacteriaceae* (*P*_adj_ = 0.0294; Figure 2C) and lower *Lactobacillaceae* (*P*_adj_ = 0.0185; Figure 2B) counts than age-matched SAMR1 mice.

**Figure 3.**
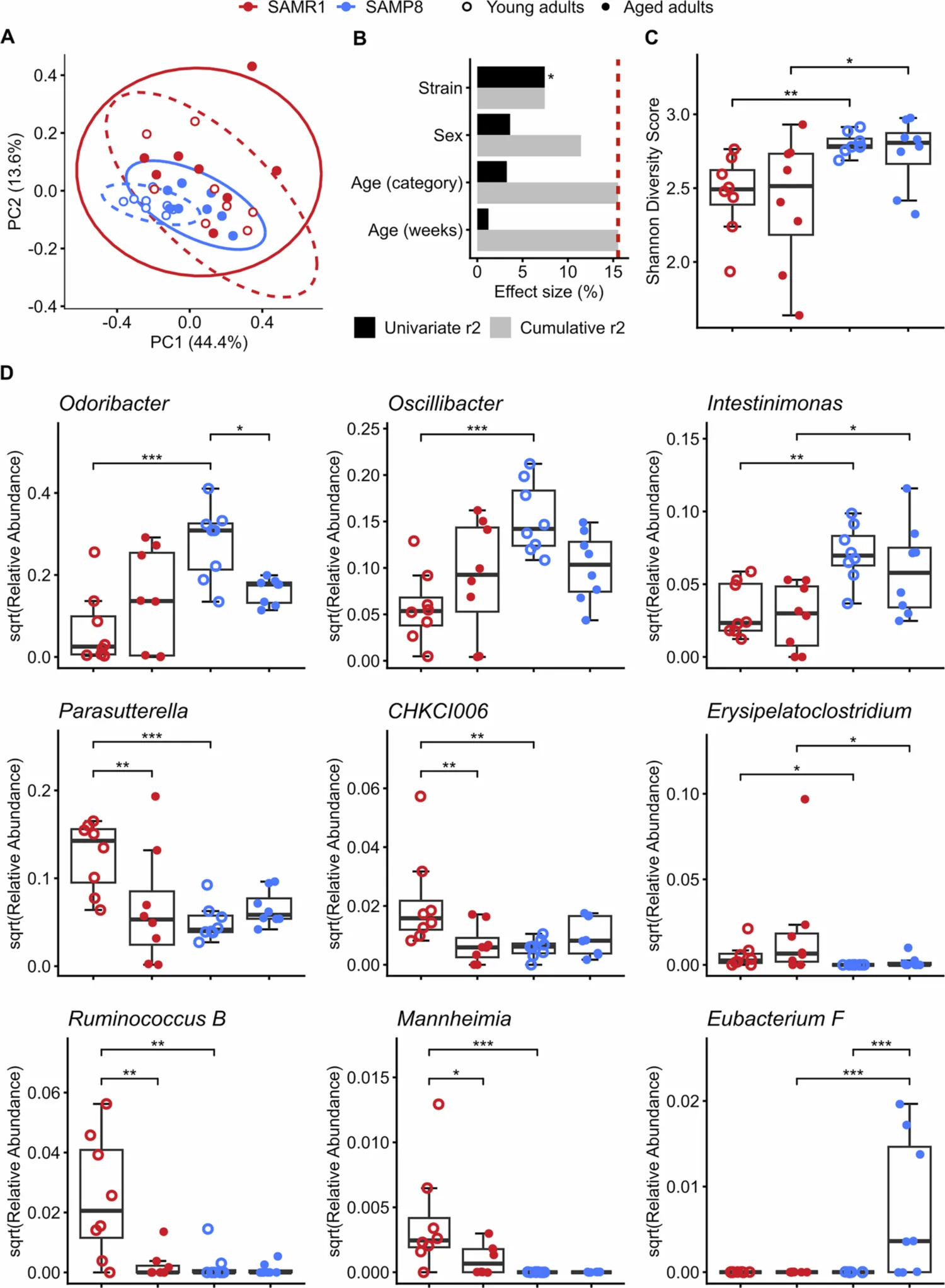
Age- and strain-related differences in gut microbiota diversity and composition as revealed by 16S rRNA sequencing. A, Principal coordinates analysis of inter-individual differences (genus-level Bray–Curtis dissimilarity) in the microbiota profiles of SAMP8 and SAMR1 mice (*n* = 4 per sex, per genotype, per age). Ellipses represent 95% confidence intervals around the group centroids. B, Explanatory power of variables on variation in microbial composition in SAMP8 and SAMR1 mice (dbRDA, genus-level Bray‒Curtis dissimilarity), either independently (single effect sizes in black, statistical significance indicated by asterisks) or in a multivariable model (cumulative effect sizes in gray; Supplementary Table S04). The cutoff for nonsignificant redundant contribution to the multivariable model is represented by the red dashed line. C, Strain-dependent differences in microbial diversity (*n* = 8 per genotype, per age; Kruskal‒Wallis, χ^2^ = 11.37, *P*_adj_ = 0.0099; Supplementary Table S05). D, Strain- and age-dependent differences in relative abundances (square root transformed) at genus level are shown only for genera that showed significant differences between SAMP8 and age-matched SAMR1 for at least one age stage (*n* = 8 per genotype, per age; Kruskal‒Wallis (*α* = 0.1), post hoc Dunn's test, statistical significance was derived from two-sided testing and adjusted for multiple testing (*P*_adj,_ BH method); Supplementary Table S05). Trends observed for most taxa were consistent in both sexes (Supplementary Figure S01; Supplementary Table S06). *, *P* < 0.05; **, *P* < 0.01; ***, *P* < 0.001.

While culture-based analyses provide insight into viable bacterial populations under defined growth conditions, they capture only a selective fraction of the intestinal microbiota. We performed culture-independent microbiome profiling by 16S rRNA gene sequencing on fecal samples selected based on the culture-based screening above, from both male and female SAMP8 and SAMR1 mice at the young adult (14–19 weeks old) and the aged stages (33–52 weeks old) (*n* = 4 per sex, per genotype, per age; Supplementary Table S03). Assessing the explanatory power of chronological age (in weeks), age category (young adults vs. aged), strain, and sex on variation in microbial composition, strain was the only significant driver, explaining 7% of the observed variance (*n* = 4 per sex, per genotype, per age; distance-based redundancy analysis (dbRDA), R^2^_adj_ = 7%, *P*_adj_ = 0.02; Figure 3A and B; Supplementary Table S04). When incorporated into a multivariable model, both sex and age category provided additional cumulative explanatory power, with the full model accounting for 15.4% of the total variation (Figure 3B). Because the study was not designed and powered to detect sex-specific effects (*n* = 4 per sex within each genotype and age group), full sex-stratified analyses were not performed. However, identified signals were inspected post hoc for sex-specificity. We observed strain-dependent differences in microbial diversity independent of age (*n* = 8 per genotype, per age; Kruskal‒Wallis test, χ^2^ = 11.37, ε^2^_R_ = 0.37, *P* = 0.0099; Figure 3C; Supplementary Table S05), with SAMP8 mice exhibiting significantly higher microbial diversity compared to SAMR1 controls both at the young adult and aged stages. Differential abundance analysis at the genus level revealed both strain- and age-dependent differences (*α* = 0.1; Figure 3D; Supplementary Table S05). At the young adult stage, SAMP8 mice showed significantly lower abundances of *Mannheimia* (*n* = 8 per genotype, per age; post hoc Dunn, *r* = −0.94, *P* = 0.0002) compared to young SAMR1 mice. In addition, several genera showed suggestive differences at the young adult stage (*P*_adj_ < 0.1), with SAMP8 mice exhibiting lower abundances of *Parasutterella* (*r* = −0.84, *P* = 0.0008), *CHKCI006* (*r* = −0.71, *P* = 0.004), *Erysipelatoclostridium* (*r* = −0.64, *P* = 0.01) and *Ruminococcus B* (*r* = −0.76, *P* = 0.002), while displaying higher abundances of *Odoribacter* (*r* = 0.87, *P* = 0.0005), *Oscillibacter* (*r* = 0.85, *P* = 0.0006), and *Intestinimonas* (*r* = 0.73, *P* = 0.004) compared to young SAMR1 mice. In the older age group, most of these strain-dependent differences were no longer observed, except for *Intestinimonas*, which remained significantly higher, and *Erysipelatoclostridium,* which remained lower in SAMP8 mice. Notably, at the aged stage, SAMP8 mice displayed a significantly higher abundance of *Eubacterium F* (χ^2^ = 17.06, ε^2^_R_ = 0.55, *P*_adj_ = 0.0261; Figure 3D) compared to young SAMP8 mice and aged SAMR1 controls. Due to the small sample size, we verified the results with LOO sensitivity analyses—all identified associations remained significant in all iterations (Supplementary Table S05). The direction of effects was comparable between males and females for microbial diversity and most genus abundances (Supplementary Figure S01; Supplementary Table S06). Sex-specific patterns were identified for the genera *Parasutterella* and *Eubacterium F*, which exhibited higher abundances in females than males in aged SAMR1 and aged SAMP8 mice, respectively. Together, the culture-based and 16S rRNA gene sequencing analyses indicate that the accelerated-aging genotype is associated with distinct alterations in the gut microbiota already observable in young adult mice. Many of these differences diminish with advancing age, suggesting a partial convergence of the microbial communities between SAMP8 and SAMR1 mice.

### 3.2. Brain and spleen T-cell composition in accelerated aging

There is growing evidence that gut microbiota dysbiosis promotes neuroinflammation, induces immune system dysfunction, and is strongly associated with cognitive decline.^6^ ^,^ ^8^ Therefore, we first sought to characterize T-cell populations in both the brain and the spleen. In the brain, we assessed possible immune infiltration or local T-cell alterations associated with neuroinflammation, while assessing a central systemic lymphoid response and change in T-cell homeostasis in the spleen.^49^ We analyzed key T-cell subsets—CD4⁺ helper and CD8⁺ cytotoxic T cells—and assessed their expression of IL-17A and IFNγ as representative markers of proinflammatory and cell-mediated immune polarization.

**Figure 4.**
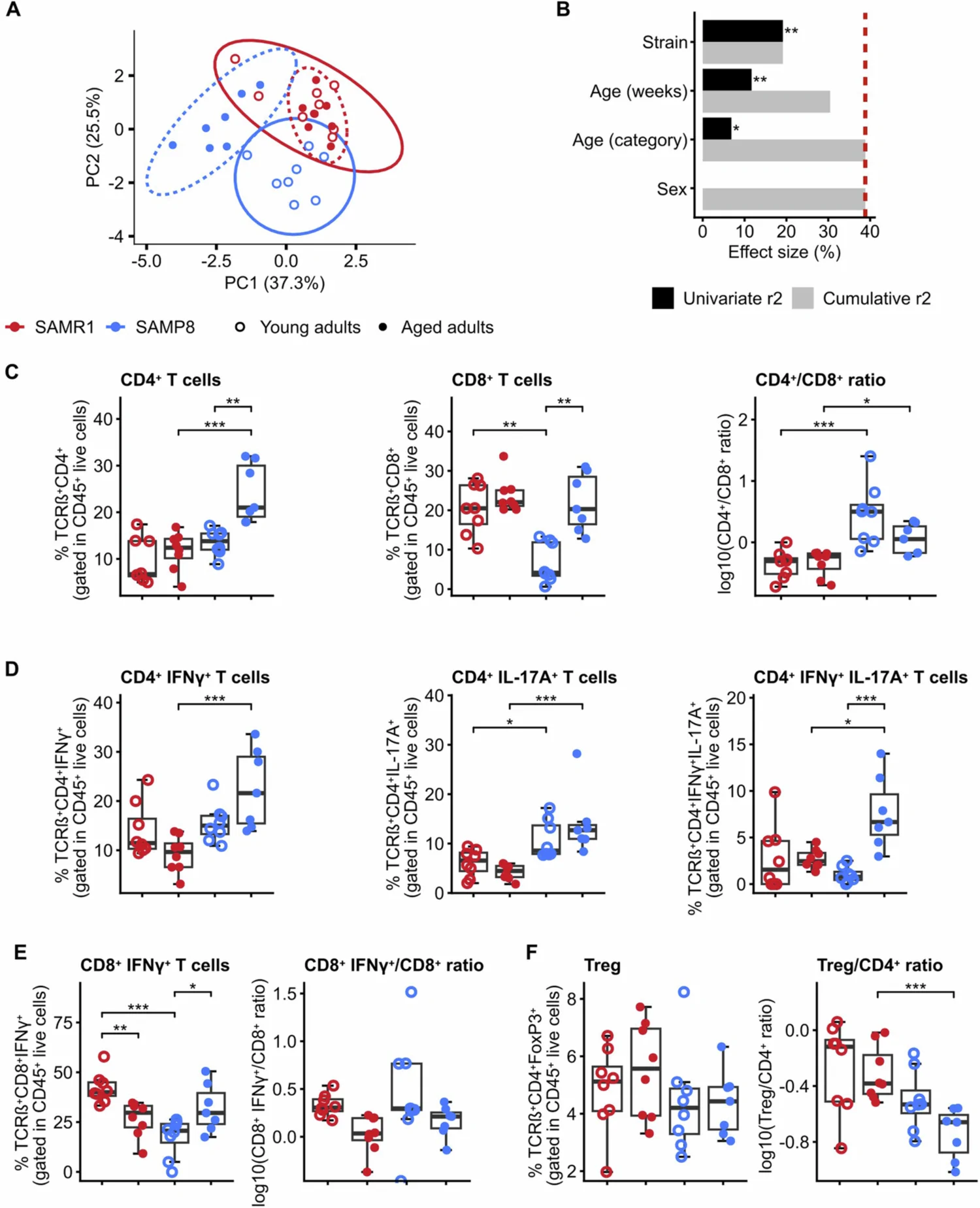
Differences in brain T-cell composition in accelerated aging. A, Principal component analysis (PCA) of interindividual differences in brain T-cell composition in SAMR1 and SAMP8 mice (*N* = 31; *n* = 8 young SAMR1, *n* = 8 aged SAMR1, *n* = 8 young SAMP8, *n* = 7 aged SAMP8; one sample was removed during FACS analysis). PCA was performed on scaled immune cell frequencies. B, Explanatory power of variables on variation in brain T-cell composition in SAMP8 and SAMR1 mice (*N* = 31; RDA, scaled immune cell frequencies, Euclidian distance; Supplementary Table S07), either independently (single effect sizes in black, statistical significance indicated by asterisks) or in a multivariable model (cumulative effect sizes in gray). The cutoff for nonsignificant redundant contribution to the multivariable model is represented by the red dashed line. C-F, Strain- and age-dependent differences in immune cell frequencies and ratios in the brain including CD4⁺ and CD8⁺ T cells and ratio (C), pro-inflammatory cytokine-producing T-cell subsets (D, E), and Tregs (F). Ratios are presented on a log10 scale for visualization. Statistical analyses were performed on non-transformed data (*N* = 31; Kruskal‒Wallis, post hoc Dunn's test; statistical significance was derived from two-sided testing and adjusted for multiple testing (P_adj_, BH method); Supplementary Table S08). Representative FACS plots are provided in Supplementary Figure S02. *, *P* < 0.05; **, *P* < 0.01; ***, *P* < 0.001.

Accelerated aging was associated with changes in both the composition and activation state of brain T cells in SAMP8 and SAMR1 controls (*N* = 31; *n* = 8 young SAMR1, *n* = 8 aged SAMR1, *n* = 8 young SAMP8, *n* = 7 aged SAMP8; one sample was removed during FACS analysis). Flow cytometry analysis revealed significant variation in brain T-cell composition across mouse strains, with strain explaining 19.2% of the observed variance (*N* = 31; redundancy analysis (RDA), R^2^_adj_ = 19.2%, *P*_adj_ = 0.004; Figure 4A and B; Supplementary Table S07; Representative FACS plots in Supplementary Figure S02). When incorporated into a multivariable model, both chronological age and age categories, but not sex, provided additional explanatory power, with the full model accounting for 39% of the total variation. More specifically, brains derived from both young and aged SAMP8 mice showed a higher CD4⁺/CD8⁺ ratio compared to those from SAMR1 controls (*N* = 31; Kruskal‒Wallis, χ^2^ = 20.7, ε^2^_R_ = 0.67, *P*_adj_ = 0.0017; Figure 4C; Supplementary Table S08). This increase was driven by distinct age-dependent changes: young SAMP8 mice showed lower frequencies of CD8⁺ T cells (post hoc Dunn, *r* = −0.73, *P* = 0.004; Figure 4C), whereas aged SAMP8 mice displayed higher levels of CD4⁺ T cells in the brain (*r* = 0.86, *P* = 0.0009; Figure 4C). Because we observed strain- and age-dependent alterations in the CD4⁺ and CD8⁺ T cells in the brain, we next examined whether these quantitative shifts were accompanied by functional polarization toward proinflammatory T-cell responses. To this end, we assessed IL-17A and IFNγ as canonical effector cytokines of Th17 and Th1/cytotoxic T-cell subsets, respectively, both implicated in neuroinflammation and age-associated immune remodeling.^16^ ^,^ ^17^ Despite comparable total CD4⁺ T cells between young SAMP8 and SAMR1 mice, brains from young SAMP8 exhibited increased levels of CD4⁺ IL-17A⁺ T cells (*r* = 0.51, *P* = 0.04; Figure 4D). Levels of CD8⁺ IFNγ⁺ T cells were reduced in brains from young SAMP8 mice, but the CD8⁺ IFNγ⁺/CD8⁺ ratio remained unchanged (Figure 4E). Brains from aged SAMP8 displayed increased levels of CD4⁺ IL-17A⁺ T cells, CD4⁺ IFNγ⁺ T cells, and CD4⁺ IFNγ⁺ IL-17A⁺ T cells compared to brains from aged SAMR1 controls (*r* = 0.996 (*P* = 0.0001), 0.97 (0.0002), and 0.52 (0.046), respectively; Figure 4D). At the young adult stage, no significant strain-dependent differences were observed regarding the levels of Tregs or Treg/CD4⁺ T-cell ratio (Figure 4F). In contrast, brains from aged SAMP8 mice exhibited a reduced Treg/CD4⁺ T-cell ratio compared to brains from aged SAMR1 controls (*r* = −0.87, *P* = 0.0008; Figure 4F). For all observed genotype- and age-dependent T-cell differences in the brain, trends were consistent across both sexes (Supplementary Table S09). Together, these findings indicate that the accelerated aging phenotype is associated with a shift towards a more pro-inflammatory immune profile in the brain, which is already detectable in young adult mice and becomes more pronounced with advancing age.

We next assessed T-cell composition and activation in the spleen to evaluate the extent to which peripheral immune changes correspond to those observed in the brain. Flow cytometry analysis showed significant variation in splenic T-cell composition with chronological age and age category explaining the highest proportion of the observed variance (*N* = 31; RDA, R^2^ = 17.2% (*P*_adj_ = 0.001) and 15.3% (0.001), respectively; Supplementary Figure S03; Supplementary Table S10; representative FACS plots in Supplementary Figure S04). Strain and sex contributed less to the observed variation (R^2^ = 12.9% (*P*_adj_ = 0.001) and R^2^ = 4.6% (*P*_adj_ = 0.021), respectively). The multivariable model including chronological age, strain, and sex accounted for 33% of the total variation. In contrast to the brain, spleens derived from SAMP8 mice exhibited a lower CD4⁺/CD8⁺ ratio compared to those from SAMR1 controls (*N* = 31; Kruskal‒Wallis, χ^2^ = 22.2, ε^2^_R_ = 0.72, *P*_adj_ = 0.002; Supplementary Figure S04; Supplementary Table S08). Increased T-cell activation, assessed by effector/naïve CD4⁺ and CD8⁺ T-cell ratios, was observed only in spleens from aged SAMP8 mice (*r* = 0.60 (*P* = 0.02), 0.53 (0.04), respectively) compared to those from young SAMP8 mice. Similarly, the pro-inflammatory cytokine-producing T cells subsets CD4⁺ IFNγ⁺ IL-17A⁺ and CD8⁺ IFNγ⁺ were increased only in spleens from aged SAMP8 mice (χ^2^ = 11.9 (ε^2^_R_ = 0.38, *P*_adj_ = 0.02), 11.0 (0.35, 0.02), respectively). By contrast, CD4⁺ IFNγ⁺ T cells were elevated in spleens from both aged SAMP8 and aged SAMR1 mice (χ^2^ = 16.8, ε^2^_R_ = 0.54, *P*_adj_ = 0.004) compared to the young adult groups, while no differences were found in splenic CD4⁺ IL-17A⁺ T cells. No significant strain- or age-dependent differences were observed in Treg frequencies. However, the Treg/CD4⁺ T-cell ratio was increased at both ages in spleens derived from SAMP8 mice compared to that of SAMR1 (χ^2^ = 16.1, ε^2^_R_ = 0.52, *P*_adj_ = 0.004). The genotype- and age-dependent differences observed in splenic T cells were consistent across both sexes (Supplementary Table S09). These results indicate that age induces a shift towards a more pro-inflammatory immune profile also in the spleen, which is more pronounced in SAMP8 mice.

### 3.3. Linking gut dysbiosis to T-cell immunity

Next, we investigated whether changes in the gut microbiota are associated with shifts in T-cell populations in the brain and spleen. T cells are central mediators of adaptive immunity through both cytotoxic and immunoregulatory functions and are strongly influenced by microbial signals that shape their differentiation and effector profiles beyond the gut environment.^50^ Microbiota-primed T cells can traffic to peripheral organs and the CNS, where they contribute to inflammatory processes along the microbiota-immune-brain axis.^24^ Furthermore, commensal microbiota shape the immune structure and cell composition of the spleen,^51^ ^,^ ^52^ supporting the concept of a gut–spleen axis in systemic immunomodulation.^53^

**Figure 5.**
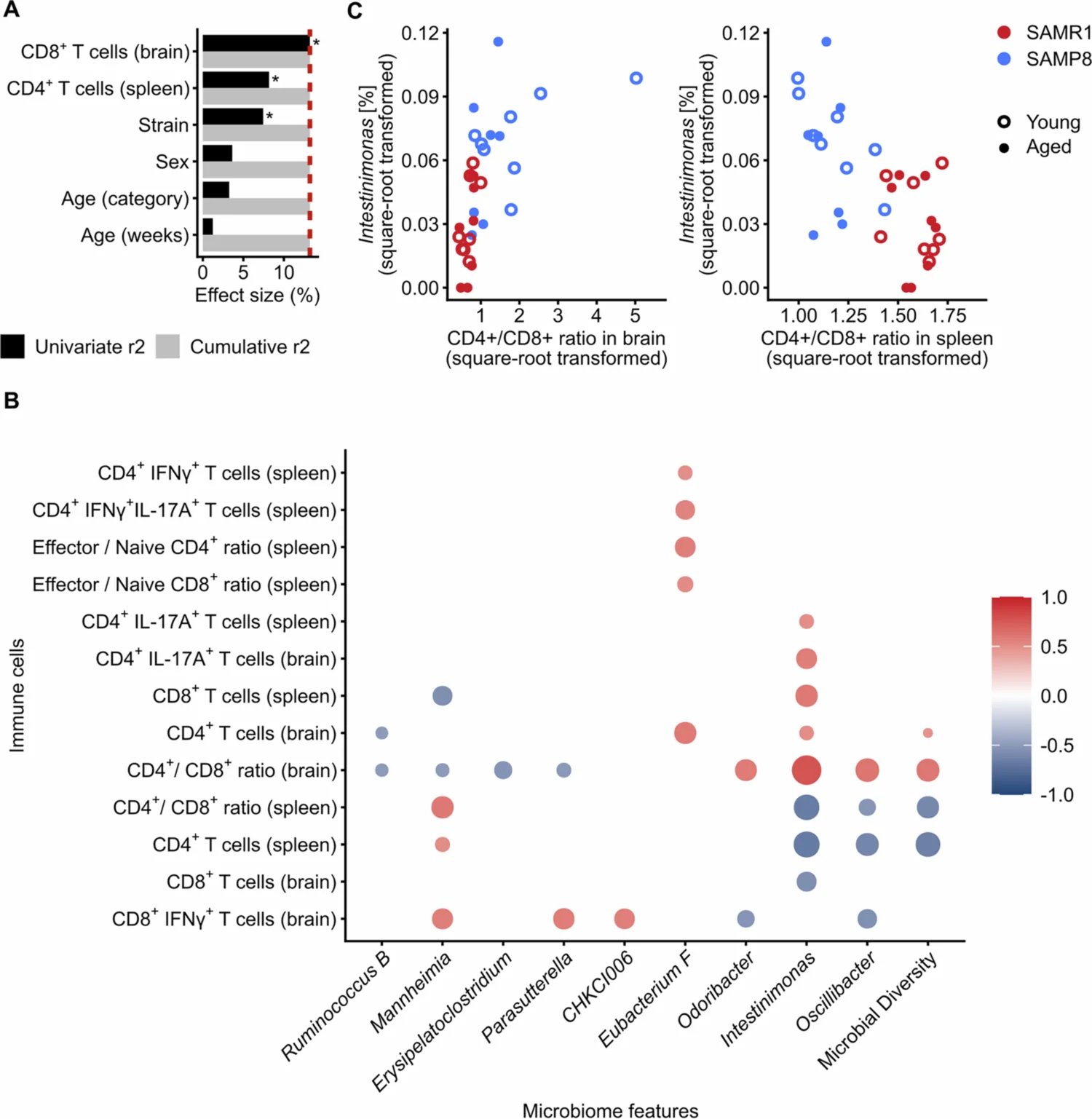
Microbial features are linked to immune signals. A, Explanatory power of variables on variation in microbial composition in SAMP8 and SAMR1 mice (*N* = 31; *n* = 8 young SAMR1, *n* = 8 aged SAMR1, *n* = 8 young SAMP8, *n* = 7 aged SAMP8; dbRDA, genus-level Bray‒Curtis dissimilarity; Supplementary Table S11), either independently (single effect sizes in black, statistical significance indicated by asterisks) or in a multivariable model (cumulative effect sizes in gray). The cutoff for nonsignificant redundant contribution to the multivariable model is represented by the red dashed line. B, Correlations between immune cell frequencies and microbiota features. Spearman correlations were computed between all immune cell frequencies and ratios and microbiota features that were identified in preceding analysis. The figure shows a selected subset of immune cell populations highlighted in the results, whereas significant correlations involving all immune cells are shown in Supplementary Figure S05. Features are ordered by hierarchical clustering (*N* = 31; Spearman correlations; Supplementary Table S12). C, *Intestinimonas* positively correlates with the CD4⁺/CD8⁺ ratio in the brain (*N* = 31; Spearman correlation, *ρ* = 0.77, *P*_adj_ < 0.001) but negatively correlates with the CD4⁺/CD8⁺ ratio in the spleen (*ρ* = −0.66, *P*_adj_ = 0.006). Values shown in the plots are square-root transformed for visualization only. Statistical significance for Spearman correlations was derived from two-sided tests using untransformed data and adjusted for multiple testing (*P*_adj_, BH method). *, *P* < 0.05; **, *P* < 0.01; ***, *P* < 0.001.

Associations between gut microbial features and specific immune cell populations were observed, with the strongest links involving brain T-cell subsets. Distance-based redundancy analysis identified the brain CD8⁺ cell frequency as significantly associated with the observed variation in microbial composition (*N* = 31; dbRDA, R^2^ = 13.2%, *P*_adj_ = 0.04; Figure 5A; Supplementary Table S11). Although frequency of splenic CD4⁺ T cells was also significantly associated with community variation (R^2^ = 8.2%, *P*_adj_ = 0.04), only brain CD8⁺ T cells remained significant when incorporated into a multivariable model including strain, age, and sex, rendering strain effects redundant. Correlation analyses further supported a relationship between gut microbial features and tissue-specific T-cell profiles (*N* = 31; Spearman; Figure 5B; Supplementary Figure S05; Supplementary Table S12). Microbial diversity was positively associated with the CD4⁺/CD8⁺ ratio in the brain but negatively associated with the CD4⁺/CD8⁺ ratio in the spleen (*ρ* = 0.62 (*P*_adj_ = 0.02) and −0.60 (*P*_adj_ = 0.02), respectively; Figure 5B). The same pattern was observed for the relative abundance of *Intestinimonas* (*ρ* = 0.77 (*P*_adj_ < 0.001) and −0.66 (*P*_adj_ = 0.006), respectively; Figure 5B and C) and *Oscillibacter* (*ρ* = 0.63 (*P*_adj_ = 0.02) and −0.53 (*P*_adj_ = 0.03), respectively; Figure 5B). *Odoribacter* correlated positively with CD4⁺/CD8⁺ ratio in the brain (*ρ* = 0.59, *P*_adj_ = 0.02), whereas *Ruminococcus B, Mannheimia*, *Erysipelatoclostridium*, and *Parasutterella* were negatively associated with the CD4⁺/CD8⁺ ratio in the brain (*ρ* = −0.50 (*P*_adj_ = 0.04), −0.50 (*P*_adj_ = 0.04), −0.54 (*P*_adj_ = 0.03), and −0.51 (*P*_adj_ = 0.04), respectively). We identified several bacteria that were linked to pro-inflammatory cytokine-producing T-cell subsets. Notably, the relative abundance of *Intestinimonas* correlated positively with CD4⁺ IL-17A⁺ T cells in both the brain and the spleen (*ρ* = 0.58 (P_adj_ = 0.016) and 0.51 (P_adj_ = 0.04), respectively; Figure 5B and C). The relative abundance of *Eubacterium F* was positively associated with T-cell activation and pro-inflammatory cytokine-producing T-cell subsets in the spleen, including CD4⁺ IFNγ⁺ IL-17A⁺ T cells (*ρ* = 0.56, *P*_adj_ = 0.02; Figure 5B). Several other bacteria correlated positively (*Mannheimia*, *Parasutterella*, and *CHKCI006*) or negatively (*Oscillibacter* and *Odoribacter*) with CD8⁺ IFNγ⁺ T cells in the brain only (Figure 5B). Due to the small sample size, we verified the results with LOO sensitivity analyses—all identified correlations remain significant in all iterations (Supplementary Table S12). Extrapolation of functional potential from phylogeny (PICRUSt2) suggested the highest abundance of core metabolic pathways, including the tricarboxylic acid (TCA) cycle and heme biosynthesis, in young adult SAMR1 mice (Supplementary Table S13), whereas a higher abundance of predicted sulfate assimilation pathways was found in young SAMP8 mice. Notably, heme biosynthesis pathways were primarily associated with *Parasutterella*, whereas sulfate assimilation pathways were most strongly linked to *Odoribacter* (Supplementary Table S14). Heme biosynthesis pathways and sulfate assimilation pathways were, respectively, negatively and positively associated with brain CD4^+^/CD8^+^ ratio (Supplementary Figure S06, Supplementary Table S14). In addition, the *N*-acetylneuraminate degradation pathway, involved in mucus degradation, was the only predicted pathway to be found in higher abundance in aged mice compared to young adults, independently of the genotype (Supplementary Table S13). These findings identify gut microbial features that are associated with brain T-cell composition and activation, implying that altered microbial communities could influence pro-inflammatory T-cell responses, thereby contributing to accelerated brain aging.

## 4. Discussion

In our study, culture-based analyses together with 16S rRNA gene sequencing identified genotype-dependent alterations in the gut microbiota. Microbiota composition variation was most strongly associated with mouse strain, with additional contributions from age and sex, whereas microbial diversity remained consistently higher in SAMP8 mice than in SAMR1 controls. Several bacterial genera displayed altered relative abundances in young SAMP8 mice, but only *Intestinimonas* remained consistently increased in aged SAMP8 mice. Immune profiling revealed strain- and age-specific T-cell signatures: brains derived from young SAMP8 mice had reduced CD8⁺ T cells, whereas brains derived from aged SAMP8 mice showed increased CD4⁺ T cells, resulting in elevated CD4⁺/CD8⁺ ratios and greater pro-inflammatory CD4⁺ T-cell responses at both ages. In contrast, spleens of SAMP8 mice exhibited reduced CD4⁺/CD8⁺ ratios, with aged mice displaying heightened T-cell activation and cytokine production. Correlation analyses linked specific bacterial genera to immune alterations, with *Intestinimonas* being negatively associated with the brain CD4⁺/CD8⁺ ratio but positively associated in the spleen. In addition, CD8⁺ T-cell frequency was significantly associated with the variation in microbiota composition.

### 4.1. Gut microbiota dysbiosis in accelerated aging

The culture-based analyses and 16S rRNA sequencing were designed to provide complementary insights into the gut microbiota. Culture-based methods quantify viable bacteria capable of growth under defined conditions, whereas 16S rRNA gene sequencing is culture-independent, representing the dominant fractions of the community. Both approaches indicated that genotype-associated microbiota alteration emerges early in adulthood and partially converges with age. The culture-based analyses further identified age-related shifts in viable *Lactobacillaceae* and *Enterobacteriaceae* populations, whereas 16S sequencing identified broader changes in community structure and genus-level composition. The increased abundance of viable *Enterobacteriaceae* together with reduced *Lactobacillaceae* observed in aged SAMP8 mice is consistent with age-associated changes reported in previous SAMP8 studies, including reductions in *Lactobacillaceae* ^54^ and expansion of Proteobacteria,^55^ the phylum containing *Enterobacteriaceae.* Both are typically considered compositional shifts in a more pro-inflammatory intestinal environment.^56^

In our study, microbial alpha-diversity, reflecting within-sample richness and evenness (quantified by using the Shannon index), was higher in SAMP8 mice than in SAMR1 controls across both age groups. This pattern was observed in both male and female mice. In contrast, previous reports assessing Shannon diversity described reduced or unchanged diversity in SAMP8 males at 5–7 months of age relative to SAMR1 controls.^57-59^ These discrepancies may be attributable to differences in the ages evaluated across studies. Consistent with our findings that microbial diversity did not differ between the two age groups, a longitudinal study in SAMP8 males reported no decline in Shannon diversity between 3 and 10 months of age.^55^ The strain-dependent differences we observed for microbial diversity were confirmed by our results on the community structure. The variation in gut microbial composition was predominantly driven by mouse strain, with age and sex contributing additional but smaller effects in multivariable models.

Microbial alterations in SAMP8 mice were characterized by a significant increase in *Intestinimonas*, a relatively recently described bacterial genus comprising at least three species that primarily colonize the intestine in both humans and mice.^60^ The most abundant species *Intestinimonas butyriciproducens* has been shown to produce butyrate from both sugars and amino acids, while the others remain poorly characterized.^60^ In the present study, taxonomic resolution was limited to the genus level, so no species-specific or functional conclusions could be drawn. The health implications of this taxon appear to be context-dependent. In a human cohort, *Intestinimonas* was linked to favorable metabolic profiles, and it was reported to improve metabolic health in one diet-induced obesity mouse model.^61^ Reduced abundance of this genus was also associated with ischemic stroke subtypes^62^ and fatigue severity during active inflammatory bowel disease in humans.^63^ In contrast, enrichment of *Intestinimonas butyriciproducens* was described to be detectable in Parkinson's disease patients with impulse control disorder (ICD), compared to those without ICD.^64^ Moreover, the genus was identified to be stress-related in a rodent model,^65^ with psychological stress leading to increased *Intestinimonas* abundance as well as increased alpha-diversity. SAMP8 mice are also reported to exhibit altered anxiety-like behaviors that vary depending on the behavioral tasks,^66^ ^,^ ^67^ which may underlie the observed association with increased *Intestinimonas* abundance. Overall, our findings suggest a potential immunological relevance of these alterations in the context of brain aging.

We further detected higher abundances of *Oscillibacter* and *Odoribacter* and lower abundance of *Parasutterella*, among others, in young SAMP8 mice compared with young SAMR1 mice. These patterns were observed in both male and female animals. However, strain-associated differences in bacterial abundances diminished with advancing age. Consistent with our observations in young mice, Peng et al.^57^ reported increased *Odoribacter* and decreased *Parasutterella* abundances in 6-month-old SAMP8 males, suggesting that strain-associated differences remain at 6 months of age. In contrast, Zhan et al.^59^ found lower *Oscillibacter* abundance in 7-month-old SAMP8 males. They also showed that fecal transplants from SAMP8, but not from SAMR1, increased the abundance of *Parasutterella* in pseudo-germ-free mice. These discrepancies may reflect age-dependent microbiota remodeling. Indeed, in our cohort, most strain-associated differences in bacterial abundances converged at older age, which is consistent with a previous report describing dynamic microbiota trajectories in SAMP8 mice.^55^ Ke et al.^55^ demonstrated distinct community structures between young (1 and 3 months) and old (7 and 10 months) SAMP8 males, highlighting substantial remodeling during aging. Such microbial remodeling could be functionally linked to early metabolic alterations.^57^ ^,^ ^68^ In our study, predictive functional profiling further suggested that the age- and genotype-dependent taxonomic differences were accompanied by differences in the predicted metabolic potential of the gut microbiota. However, as computational extrapolations are based on phylogenetic information, these functional predictions should be interpreted with caution. Peng et al.^57^ associated gut bacterial perturbations in SAMP8 mice with dysregulated lipid, carbon, and pyruvate metabolism, supporting a connection between microbiota composition and host metabolic pathways. Expanding on this functional perspective, Dacomo et al.^68^ observed that differential gene expression in SAMP8 was limited to younger age groups (2.5 and 6 months) and metabolomic alterations in the plasma were strongest at 2.5 months, attenuating thereafter. In particular, SAMP8 mice showed lower plasma levels of acylcarnitines and several lipid species compared to age-matched SAMR1 controls, correlating with various neuropathology indicators.^68^ These findings support that accelerated aging in SAMP8 mice is associated with pronounced perturbations of gut microbial communities during early adulthood. Such perturbations may influence host metabolic and immune pathways, potentially contributing to later neuropathological phenotypes. However, at a more advanced age, other physiological aspects of chronological age may become dominant driver(s) shaping the gut microbiota and thus outweigh the differences between SAMP8 and SAMR1 mice.

### 4.2. Immune dysregulation in SAMP8 mice

Age- and strain-dependent differences in brain T-cell profiles suggest distinct immunosenescence trajectories in SAMP8 mice. We observed a higher CD4⁺/CD8⁺ ratio in the brains of both young and aged SAMP8 mice compared to age-matched SAMR1 controls, driven by reduced CD8⁺ T-cell frequencies in young SAMP8 and increased CD4⁺ T cells in aged SAMP8 brains. To our knowledge, no previous studies have reported comparable analyses of whole brain tissue. In the absence of directly comparable datasets, we examined related evidence from human peripheral blood studies.^69^ ^,^ ^70^ The latter studies demonstrated age-associated immune shifts in the same direction as those observed in our mouse model. Specifically, peripheral blood immune profiling of 363 Taiwanese individuals stratified by age showed significantly decreased CD8⁺ T cells and increased CD4⁺/CD8⁺ ratios. In contrast, absolute CD4⁺ T-cell numbers remained stable, although reversed naïve/memory trends were observed with age.^69^ These findings were further supported by a meta-analysis of 12 human studies comprising 7,425 adults across age groups.^69^ The analysis confirmed declining CD8⁺ T cells, rising CD4⁺/CD8⁺ ratios and Natural Killer (NK) cells, and relatively stable CD4⁺ T-cell numbers, with linear regression demonstrating consistent correlations with age.^69^ Additionally, a study of 92 healthy individuals (ages 3–88 y) similarly reported a marked age-associated decline in CD8⁺ T cells accompanied by an increased CD4⁺/CD8⁺ ratio.^70^ These data closely parallel our observations in SAMP8 mice, in which accelerated aging increases the CD4⁺/CD8⁺ ratio. This suggests that SAMP8 mice may represent a pathological exaggeration of normal aging-related immunosenescence, supporting the translational relevance of this model. Furthermore, pro-inflammatory CD4⁺ responses were enhanced in both young and aged SAMP8 brains, with exacerbated inflammatory T-cell phenotypes observed in aged SAMP8 mice. Si et al.^71^ used scRNA-seq (18-month-old vs. 10-week-old female C57BL/6 mice, *n* = 4 per group) and demonstrated that aging increases CD4⁺ and CD8⁺ T-cell abundance in mouse brain border tissues (meninges and choroid plexus) and the hippocampus. Aging drove a shift toward an effector memory phenotype, characterized by depletion of naïve (Sell⁺ CD44^-^) cells and accumulation of cytotoxic CD8⁺ T cells (expressing high levels of cytotoxic molecules such as *Gzmb* (granzyme B) and *Gzmk* (granzyme K) and cytokines including *Ifng* (interferon gamma) and *Tnf* (tumor necrosis factor)) in the meninges.^71^ They also observed increases in distinct effector CD4⁺ T-cell populations, including CD153⁺ CD4⁺ T cells (expressing the TNF superfamily gene *Tnfsf8*), IL2-expressing effector CD4⁺ T cells co-expressing *Ifng*, and Tregs in the meninges and choroid plexus, as well as CD153⁺ CD4⁺ T cells in the hippocampus.^71^ Complementing this, Rousseau et al.^72^ detailed the underlying epigenetic mechanisms: aged CD8⁺ T cells exhibit chromatin closure (hypermethylation of *CCR7*, *CD27*) suppressing cytotoxicity, while CD4⁺ cells show *IFNγ* promoter hypomethylation driving Th1 skew and Treg dysfunction amid metabolic stress. The reduction of CD8⁺ T cells in young SAMP8 mice parallels epigenetic silencing of activation-associated genes, whereas the expansion of IFNγ⁺ and IL-17A⁺ CD4⁺ T cells in aged SAMP8 mice is consistent with Th1/Th17 skewing driven by pro-inflammatory epigenetic reprogramming. Regulatory oversight remained intact at the young adult stage, with no strain-dependent differences in Tregs or Treg/CD4⁺ ratios, but aged SAMP8 brains showed a marked reduction in the Treg/CD4⁺ ratio, signaling immune exhaustion and failure to suppress effector responses. Consistent with animal evidence, De la Fuente et al.^73^ observed a nonsignificant trend in brain Tregs but reported increased tissue-resident (CD69⁺) Tregs in the aged brain. Si et al.^71^ noted increased Tregs in the meninges and choroid plexus, highlighting regional differences in regulatory populations. These findings integrate with the established SAMP8 phenotype of early glial activation, tau hyperphosphorylation, synaptic loss, and neuroinflammation.^26^ Early CD8⁺ T-cell deficits may permit these changes to accumulate, while aged Th1/Th17 polyfunctionality and Treg collapse may further accelerate their progression, paralleling peripheral-to-CNS immune trafficking in neurodegeneration. Under physiological conditions, the CNS parenchyma is largely devoid of conventional T cells, with Treg cells primarily confined to border regions such as the meninges and choroid plexus.^74^ As the meninges and choroid plexus were not removed during CNS processing in the present study, the detected T-cell populations likely represent a combination of parenchymal and border-associated immune cells. Nevertheless, the observed qualitative changes in T-cell composition are consistent with inflammation-associated immune recruitment driven by blood–brain barrier (BBB) dysfunction and neuroimmune activation.^75^ ^,^ ^76^ In this context, early CD8⁺ T-cell reduction in young SAMP8 mice may indicate altered immune surveillance dynamics at CNS interfaces. In aged SAMP8 mice, the expansion of IFNγ⁺ and IL-17A⁺ CD4⁺ T cells, together with reduced Treg/CD4⁺ ratios, suggests sustained inflammatory trafficking and impaired immune regulation once BBB integrity declines.^77^ ^,^ ^78^ Collectively, these data suggest that aging promotes shifts in brain-associated T-cell populations toward inflammatory phenotypes, which may exacerbate neuroinflammatory cascades and contribute to an accelerated aging phenotype in SAMP8 mice.

Moreover, splenic immune profiling revealed evidence of accelerated peripheral inflammaging in SAMP8 mice. Aging exacerbated T-cell remodeling selectively in SAMP8 mice, as indicated by increased effector-to-naïve CD4⁺ and CD8⁺ T-cell ratios. Similar to our findings, scRNA-seq data from splenic CD4⁺ T cells of young (2–3 months; *n* = 4) and old (22–24 months; *n* = 4) healthy mice revealed a significant reduction in naïve cells (CD4⁺CD62L⁺CD44^-^) and an increase in effector-memory cells (CD4⁺CD44⁺CD62L^-^) in old mice.^79^ Cells overexpressing *Eomes* (Eomesodermin), *Gzmk,* and *Ctla2a* (cytotoxic T lymphocyte-associated protein 2 alpha), genes associated with CD8⁺ cytotoxic T-cell subsets, were highly enriched in aged mice.^79^ Such a shift toward differentiated effector phenotypes is characteristic of age-associated contraction of the naïve T-cell pool and persistent antigenic stimulation.^80^ ^,^ ^81^ Functionally, this remodeling in aged SAMP8 mice was accompanied by a selective expansion of pro-inflammatory T-cell subsets, including CD4⁺ IFNγ⁺ IL-17A⁺ and CD8⁺ IFNγ⁺ cells. The age-associated increase in splenic Th1 polarization (CD4⁺ IFNγ⁺) observed in both aged SAMP8 and SAMR1 mice aligns with the concept of inflammaging, characterized by chronic low-grade inflammation during aging.^82^ Notably, the emergence of dual IFNγ⁺ IL-17A⁺ CD4⁺ T cells and heightened CD8⁺ IFNγ⁺ responses exclusively in aged SAMP8 animals suggests an amplified inflammatory bias beyond that seen in normal aging. Furthermore, although total Treg frequencies were unchanged in SAMP8 mice, the increased Treg/CD4⁺ T-cell ratio likely reflects contraction of the conventional CD4⁺ compartment rather than true Treg expansion. Age-related studies indicate that Treg numbers are often preserved or modestly increased during aging, but their suppressive efficiency may be altered.^79^ ^,^ ^83^ ^,^ ^84^ The relative Treg/CD4⁺ enrichment may represent a compensatory, yet insufficient, response to heightened inflammatory activation. Collectively, these findings indicate that SAMP8 mice exhibit baseline immune alterations followed by age-dependent amplification of effector differentiation and pro-inflammatory cytokine production. This peripheral immune acceleration may contribute to systemic inflammatory burden and could mechanistically intersect with tissue-specific aging processes observed in this model.

### 4.3. Linking gut dysbiosis and T-cell subtype abundance to brain health

Sex is known to influence both gut microbiota composition and immune function, including T-cell responses, in mice and humans,^85^ ^,^ ^86^ suggesting that sex may alter host‒microbiome interactions in aging. In this study, both male and female mice were included. Overall, genotype and/or age explained a substantially larger proportion of the observed variation in microbiota composition as well as T-cell composition in the brain and spleen than sex. Moreover, the direction of genotype and age-associated effects was generally consistent between males and females. Nevertheless, two genera exhibited apparent sex-specific patterns—*Eubacterium F* and *Parasutterella*. These observations warrant confirmation in larger studies designed to investigate sex-dependent host‒microbiome interactions in the aging context. In contrast, no sex-specific patterns were observed for T cells in the brain and spleen.

Our data reveal that gut microbial composition is intricately linked with systemic and central immune profiles in a tissue‑specific manner, supporting an emerging model of microbiota‒immune‒brain crosstalk that may influence cognitive health. We observed that overall microbial diversity was positively correlated with the CD4⁺/CD8⁺ ratio in the brain while negatively correlated with the ratio in the spleen. This suggests that gut microbiota diversity differentially shapes T-cell distribution between the CNS and peripheral immune compartments. Specific taxa displayed strikingly divergent associations. For example, *Intestinimonas* and *Oscillibacter* were positively associated with brain CD4⁺/CD8⁺ ratios yet negatively with the one in the spleen, while *Odoribacter* was positively associated only with the brain CD4⁺/CD8⁺ ratio. These patterns align with clinical observations showing that members of the *Odoribacteraceae* family are enriched in individuals with cognitive impairment and associated with altered brain structural metrics and cognitive decline in a human cohort.^87^ Altered gut microbiota community diversity and specific compositional shifts are seen in the SAMP8 mouse line. These microbiota changes are associated with impaired spatial learning and memory. In addition, fecal microbiota transplantation (FMT) from controls partially restores cognitive performance and alpha-diversity in recipient mice.^59^ ^,^ ^88^ These findings support the concept that dysbiotic microbiota contribute to age-related neurodegenerative processes reported in this model. In the SAMP8 mouse model, early cognitive impairment occurs together with increased pro-inflammatory cytokine production, astrocytosis, microgliosis, tau hyperphosphorylation, neuronal loss, and reduced neurogenesis during aging, consistent with gut–brain-driven neurodegenerative changes observed between 3 and 6 months of age.^27^

Experimental manipulation of gut microbiota further supports its potential contribution in neuroinflammation, immune homeostasis, and brain function during aging and disease. FMT between young and aged mice has shown that the aging gut microbiota resulted in increased intestinal permeability, cytokine signaling (IL-6, TNF), microglial activation and retinal inflammation, while transfer of young microbiota can partially reverse age-associated inflammatory changes.^89^ Similarly, transplantation of aged donor microbiota into young recipients impaired spatial learning and memory and induced an aging-like microglial phenotype in the hippocampus, indicating that age-associated microbiota changes can directly affect CNS function.^90^ In addition to FMT studies, antibiotic and colonization approaches further demonstrate that the gut microbiota can shape immune phenotypes. Antibiotic-impacted microbiota has been shown to impair the development of colonic CD4⁺ Treg cells and predispose mice to dysregulated immune responses, supporting a causal role of microbiota composition in T-cell immune homeostasis.^91^ Moreover, mono-colonization with defined *Akkermansia muciniphila* strains modulated microglial and astrocyte gene expression and altered peripheral T-cell populations, including γδ-T cells and IFNγ-producing CD4⁺ T cells, suggesting that specific bacterial taxa may influence CNS immune regulation through peripheral immune and microbial metabolite pathways.^92^ Together, these studies support the concept that microbiota alterations may contribute mechanistically to neuroimmune aging. However, dedicated interventional experiments will be necessary to confirm causality in our experimental setting.

Beyond compositional differences, our correlational data linking taxa to pro‑inflammatory T-cell subsets suggest functional immunomodulatory roles. *Intestinimonas*, recognized as a bacterium with the potential for butyrate-production, was positively associated with CD4⁺ IL‑17A⁺ T cells in both the brain and spleen, whereas *Eubacterium F* was positively associated with splenic CD4⁺ IFNγ⁺ IL‑17A⁺ T cells. Although these associations do not establish causality, they are consistent with evidence that microbiota-derived metabolites, including SCFAs such as butyrate, as well as tryptophan-derived metabolites and bile acid derivatives, have been shown to regulate inflammatory and regulatory T-cell programs.^93^ Butyrate promotes regulatory T-cell differentiation through G protein-coupled receptor (GPCR) signaling and histone deacetylase (HDAC) inhibition.^94^ ^,^ ^95^ It also influences effector T-cell polarization by promoting Th1 differentiation through upregulation of T-bet while suppressing the expression of RORγt and other Th17-associated transcription factors, highlighting its complex immunomodulatory properties.^96^ In addition, it modulates CD8⁺ T-cell immunity via the GPR109A/HOPX pathway.^97^ This and other microbiota-derived metabolites may communicate with the CNS through immune, neural (including vagal), and metabolic pathways, providing a plausible mechanism linking gut microbial composition to brain immune alterations.^98^ ^,^ ^99^ Furthermore, the human pilot study by Vivek et al.^100^ conducted immunophenotyping alongside 16S rRNA sequencing and found that naïve CD4⁺ T-cell frequencies correlated with relative abundances of gut bacteria such as *Intestinimonas*, linking peripheral T-cell subsets to microbiota composition in healthy adults. The brain‑specific associations with CD8⁺ IFNγ⁺ cells observed here (e.g., positive correlations for *Mannheimia*, *Parasutterella* and negative correlations for *Oscillibacter*, *Odoribacter*) further support the concept that microbe‑specific cues may shape cytotoxic T-cell responses in the CNS. These responses may contribute to neuroinflammatory processes involved in cognitive decline. This interpretation is further supported by recent evidence that aged circulating CD8⁺ T cells and their secreted factor GZMK can impair hippocampal-dependent learning and memory, indicating that altered CD8⁺ T-cell immunity may contribute directly to age-related cognitive decline.^101^ More broadly, these findings resonate with mechanistic studies in animal models demonstrating that gut dysbiosis influences neuroinflammation and CNS immune cell infiltration. For example, experimental perturbation of gut commensal-specific CD4⁺ T cells can license their infiltration into the CNS and drive neuroinflammatory phenotypes characterized by IL-17A and IFNγ production.^24^ Together, these studies support the concept that gut microbiota can modulate both peripheral and central T-cell responses relevant to neuroinflammation. In addition, probiotic interventions have been shown to reduce bacteria‑related pattern recognition receptor signaling (e.g., TLR4, RIG‑I) and inhibit NF‑κB‑mediated inflammation in both gut and brain, leading to improved synaptic and neuronal outcomes.^25^ ^,^ ^102^ In aging and Alzheimer's disease models, dysbiosis has been implicated in driving inflammation, altering microbial metabolite profiles, and exacerbating neuropathological features such as amyloid deposition and cognitive impairment, suggesting overlapping inflammatory mechanisms between gut dysbiosis and neurodegeneration.^57^ ^,^ ^59^ ^,^ ^88^

Taken together, our results reinforce the concept that gut microbiota compositions not only correlate with peripheral immunity but can also shape central immune balances and pro-inflammatory states that are linked to cognitive impairment and neurodegenerative phenotypes in human and animal studies. The tissue-specific associations underscore the complexity of microbiota‒immune‒brain interactions and suggest that targeting gut dysbiosis might modulate both systemic and CNS immune responses, representing a potential strategy to influence neuroinflammation and cognitive trajectories in aging and disease.

### 4.4. Limitations and future perspectives

While our study provides a detailed characterization of gut microbiota dysbiosis and its association with brain T-cell immunity in accelerated aging, several limitations should be considered. First, the low number of T cells recovered from the brain samples restricted a more comprehensive phenotypic characterization of CNS-associated T-cell populations, including the analysis of naïve, effector, and central memory cells within the CD4⁺ and CD8⁺ compartments. As a consequence, the interpretation of brain immune alterations in aged mice is primarily based on total T-cell frequencies and activation-associated markers, limiting conclusions regarding the contribution of specific T-cell subsets to neuroimmune aging. Second, although mice were perfused prior to brain collection in order to reduce contamination by circulating immune cells, the meninges were not removed during CNS processing. Consequently, the isolated immune cell population may comprise both parenchymal and meningeal-associated cells, including cells from the choroid plexus. In addition, markers commonly used to discriminate tissue-resident from circulating T cells, such as CD69 and CD103, were not included in the flow cytometry panel, limiting a more precise characterization of the origin and residency status of brain-associated T-cell populations. Third, even though the culture-based assessment of microbiota composition was performed longitudinally, the 16S rRNA gene sequencing analyses and T-cell analyses were conducted cross-sectionally, using separate young and aged cohorts. As a result, the broader microbiota profiling and immune analyses remain correlative. They do not allow causal inference regarding the direction of the observed microbiota-immune associations, nor the temporal relationship between central and peripheral immune aging.

Future studies addressing these gaps could provide mechanistic insight into gut‒brain immune interactions. Optimized CNS immune cell isolation protocols, including the removal of the meninges, combined with high-dimensional flow cytometry and single-cell approaches, will enable a more comprehensive characterization of brain-associated T-cell subsets during aging and facilitate the discrimination of tissue-resident from circulating immune populations. Longitudinal studies integrating microbiome and immune analyses will be required to determine the temporal and causal relationship between peripheral and central immune aging. Functional profiling of the gut microbiome using metagenomic shotgun sequencing, combined with assessment of gut barrier integrity, characterization of intestinal immune populations and markers of brain inflammation would help clarify how microbial changes influence neuroimmune aging. Future intervention studies employing FMT, antibiotic-mediated microbiota depletion, and defined bacterial colonization approaches will be required to determine whether gut microbiota alterations drive brain T-cell changes and whether their modulation improves cognitive outcomes.

## 4.5. Conclusion

In conclusion, accelerated aging in SAMP8 mice is associated with early strain-specific alterations in gut microbiota composition accompanied by distinct remodeling of brain T-cell populations that seem to precede overt systemic immune activation. The association between microbial composition and CD4⁺ and CD8⁺ T-cell dynamics, including a strong link to *Intestinimonas*, supports a connection between gut dysbiosis and neuroimmune homeostasis. Together, these findings reinforce the concept that interactions between the microbiota and the immune system are an essential component of age-related brain immune remodeling.

## Supplementary Material

Supplementary_Material_V4.docx Supplementary_Material_V4.docx

SupplementaryTables_V3.xlsx SupplementaryTables_V3.xlsx

## Data Availability

For the primary microbiome-immune profiling cohort, the metadata and processed data to reproduce the analysis are available on Zenodo (doi: 10.5281/zenodo.19130008). Raw amplicon sequencing data that support the findings of this study have been deposited in European Nucleotide Archive with accession code PRJEB110524 with public access. The remaining data is available upon request.
