# Supplementary material for: Gut microbiota dysbiosis shapes brain T-cell immunity in accelerated aging
Source: Gut Microbes. 2026 Sep 17;18(1):2728329. doi: 10.1080/19490976.2026.2728329 (PMC13596893; doi:10.1080/19490976.2026.2728329)
Supplement: Supplementary file 1 [file KGMI_A_2728329_SM7170.docx]

Supplementary Material

**This file includes:**
Supplementary Figures 1-6
Legends of Supplementary Tables 1-14

**Other supplementary material for this manuscript:**
Supplementary Tables 1-14 (provided in one excel file)

**Supplementary Figure S01. Strain-, age-, and sex-dependent differences in microbiome features.** Microbiome features are shown stratified by sex and in the combined dataset (**A,B**). Boxplots display results for both females and males combined (All) as well as for females (F) and males (M) separately. Sex-stratified analyses revealed comparable effect directions in females and males for microbial diversity (**A**) and most genus abundances (**B**). An exception were the genera *Parasutterella* and *Eubacterium F* (**C**), which exhibited higher abundances in females than males in aged SAMR1 and aged SAMP8 mice, respectively (n = 4 per sex, per genotype, per age; Kruskal-Wallis (α = 0.1), post hoc Dunn’s test, statistical significance was derived from two-sided testing and adjusted for multiple testing (P_adj_, Benjamini-Hochberg (BH) method); Supplementary Table S06). *, p < 0.05; **, p < 0.01; ***, p < 0.001. Abbreviations: All, combined males and females; F, females; M, males.

**Supplementary Figure S02. Representative FACS plots of T cell subsets in the brain related to Figure 3.** Cells were gated on CD45⁺ live cells and analyzed for CD4⁺ and CD8⁺ T cells (**A**), CD4⁺ IFNγ⁺ and IL-17A⁺ T cells (**B**), CD8⁺ IFNγ⁺ T cells (**C**), and regulatory T cells (Tregs; **D**).

**Supplementary Figure S03. Spleen T cell composition in accelerated aging.** **A,** Principal component analysis (PCA) of inter-individual differences in spleen T cell composition in SAMR1 and SAMP8 mice (N = 31; n = 8 young SAMR1, n = 8 aged SAMR1, n = 8 young SAMP8, n = 7 aged SAMP8). PCA was performed on scaled immune cell frequencies. **B,** Explanatory power of variables on variation in spleen T cell composition in SAMP8 and SAMR1 mice (N = 31; RDA, scaled immune cell frequencies, Euclidian distance), either separately (single effect sizes in black, statistical significance indicated by asterisks) or in a multivariable model (cumulative effect sizes in grey; Supplementary Table S10). The cut-off for non-significant redundant contribution to the multivariable model is represented by the red dashed line. **C-F,** Strain- and age-dependent differences in immune cell frequencies and ratios in the spleen including CD4⁺ and CD8⁺ T cells and ratio (**C**), effector/naïve T cell ratio (**D**), pro-inflammatory cytokine producing T cell subsets (**E**) and T regulatory cells (Tregs; **F**). Ratios are presented on a log10 scale for visualization. Statistical analyses were performed on non-transformed data (N = 31; Kruskal-Wallis, Dunn’s test, statistical significance was derived from two-sided testing and adjusted for multiple testing (P_adj_, BH method); Supplementary Table S08). *, p < 0.05; **, p < 0.01; ***, p < 0.001

**Supplementary Figure S04. Representative FACS plots of T cell subsets in the spleen related to Supplementary Figure S03.** Cells were gated on CD45⁺ live cells and analyzed for CD4⁺ and CD8⁺ T cells (**A**), effector and naïve CD4⁺ T cells (**B**), effector and naïve CD8⁺ T cells (**C**), CD4⁺ IFNγ⁺ and IL-17A⁺ T cells (**D**), CD8⁺ IFNγ⁺ T cells (**E**) and regulatory T cells (Tregs; **F**).

**Supplementary Figure S05. Comprehensive correlations between T cell features and microbiome features.** Spearman correlations were computed between all T cell frequencies and ratios and microbiome features that were significant in preceding analysis. T cells and microbiome features are ordered by hierarchical clustering (N = 31; n = 8 young SAMR1, n = 8 aged SAMR1, n = 8 young SAMP8, n = 7 aged SAMP8; Spearman, statistical significance was derived from two-sided testing and adjusted for multiple testing (P_adj_, BH method); Supplementary Table S12).

**Supplementary Figure S06. Significant correlations between predicted metabolic pathways of the gut microbiota and immune cell frequencies and ratios.** Spearman correlations were computed between all immune cell features and pathway abundances that were selected in preceding analyses. Pathways and immune cells are ordered by hierarchical clustering (N = 31; n = 8 young SAMR1, n = 8 aged SAMR1, n = 8 young SAMP8, n = 7 aged SAMP8; Spearman, statistical significance was derived from two-sided testing and adjusted for multiple testing (P_adj_, BH method); Supplementary Table S14).

# Supplementary Tables Legends

**Supplementary Table S01.** Phenotypic characterization of accelerated aging in SAMP8 mice compared with SAMR1 mice. Grip strength of SAMP8 and age-matched SAMR1 mice at 3, 5, 7, and 10 months of age were measured using a Newton meter and normalized to body weight (gramforce/weight). Statistical analyses were performed using Brown-Forsythe and Welch ANOVA followed by Dunnett's T3 multiple-comparisons test (N = 336; n = 36–52, male and female for SAMR1 and SAMP8). Spatial working memory assessed by the T-maze test in 10-month-old SAMP8 and SAMR1 mice, presented as the percentage of right choice. Statistical significance was determined using an unpaired two-tailed Mann-Whitney U test (N = 17; n = 8 [3 males, 5 females] for SAMR1; n = 9 [4 males, 5 females] for SAMP8). Raw transcriptomic data of representative brain gene-expression markers associated with glial activation, neuroinflammation, and brain homeostasis are shown for male SAMP8 and SAMR1 mice at 3 and 10 months of age (n = 4 per age and genotype). Differential gene expression analysis was performed using the DESeq2 package, and differentially expressed genes were identified using a Benjamini-Hochberg false discovery rate (FDR)-adjusted P value < 0.01.

**Supplementary Table S02.** Culture-based fecal microbiota composition in SAMR1 and SAMP8 mice at 3, 5, 7 and 10 months of age (N = 335; n = 27–52, male and female for SAMR1 and SAMP8). Fecal pellets were collected at the indicated ages, plated on selective media, and quantified as colony-forming units (CFUs) per mg of feces. CFUs grown on selective agar for *Lactobacillaceae*, *Enterobacteriaceae* or on Schaedler agar. Statistical analysis was performed by Kruskal–Wallis test followed by Dunn's multiple comparisons test. Abbreviations: mo, months.

**Supplementary Table S03.** Summary of characteristics (strain, age in weeks, age as category, sex, study group) and immune cell frequencies of the mouse cohort used for 16S rRNA sequencing and FACS analyses. All analyses presented in Supplementary Tables S04-S14 were performed on this cohort.

**Supplementary Table S04.** dbRDA model association statistics calculated on gut microbiome variation (genus-level Bray-Curtis dissimilarity) in SAMR1 and SAMP8 mice (n = 8 per genotype, per age) using the variables strain, age (weeks), age (category) and sex. Explanatory power and significance of all included variables are reported using single variable dbRDA and stepwise multivariable dbRDA. Explanatory power R^2^ and R^2^ adjusted (a modified version accounting for the number of predictors) are reported as “r2” and “r2adj”, respectively. P values are adjusted for multiple testing correction using the Benjamini-Hochberg method and are reported as “P_adj_”.

**Supplementary Table S05.** Differences of fecal microbiome features (Shannon diversity and relative genus abundances) between study groups (n = 8 per genotype, per age). Kruskal-Wallis tests were performed to evaluate overall differences in the distributions of microbiome features across study groups. Statistical significance was assessed using two-sided tests with a significance threshold of α = 0.1, and p-values were adjusted for multiple testing across all features using the Benjamini-Hochberg method (P_adj_). For significant results, leave-one-out sensitivity analyses were performed by recalculating Kruskal-Wallis tests iteratively after removing one sample at a time. The percentage of iterations for which the results remained significant are reported as “% sign.”. When the Kruskal-Wallis test was significant, post-hoc Dunn’s tests were conducted to assess pairwise differences between study groups. Effect sizes for Kruskal-Wallis tests were reported as ordinal epsilon squared (ε^2^_R_), calculated from the test-derived H-statistic divided by sample size minus one, and effect sizes for Dunn’s tests were reported as “r”, calculated as the test-derived Z-statistic divided by the square root of sample size. “N” indicates the total number of samples for the Kruskal-Wallis test. “N (pairwise)” indicates the combined sample size of the two groups being compared in each post hoc Dunn’s test.

**Supplementary Table S06.** Differences of fecal microbiome features (Shannon diversity and relative genus abundances) between study groups in female mice and male mice separately, and across all study groups including both sexes (n = 4 per sex, per genotype, per age). Only microbiome features that were identified as significant in the Kruskal-Wallis test performed across all samples (males and females combined; see Supplementary Table S05) were included in this sex-stratified analysis. Kruskal-Wallis tests were conducted to evaluate overall differences in the distributions of the selected microbiome features across the four female study groups, the four male study groups, and all eight study groups combined. Statistical significance was assessed using two-sided tests with a significance threshold of α = 0.1, and p-values were adjusted for multiple testing across all features using the Benjamini-Hochberg method (P_adj_). When the Kruskal-Wallis test was significant, post-hoc Dunn’s tests were conducted to assess pairwise differences between study groups. Effect sizes for Dunn’s tests were reported as “r”, calculated as the test-derived Z-statistic divided by the square root of sample size. “N” indicates the total number of samples for the Kruskal-Wallis test. “N (pairwise)” indicates the combined sample size of the two groups being compared in each post hoc Dunn’s test. The first and second sections of this table present the female-only and male-only analyses, respectively, whereas the third section presents the combined eight-group analysis, including female-versus-male comparisons within genotype- and age-matched study groups.

**Supplementary Table S07.** RDA model association statistics calculated on the brain T cell composition variation (Euclidean distance) in SAMR1 and SAMP8 mice (N = 31; n = 8 young SAMR1, n = 8 aged SAMR1, n = 8 young SAMP8, n = 7 aged SAMP8) using immune cell frequencies (scaled). Explanatory power and significance of all included variables are reported using single variable RDA and stepwise multivariable RDA. Explanatory power R^2^ and R^2^ adjusted (a modified version accounting for the number of predictors) are reported as “r2” and “r2adj”, respectively. P values are adjusted for multiple testing correction using the Benjamini-Hochberg method and are reported as “P_adj_”.

**Supplementary Table S08.** Differences of T cell frequencies and ratios between study groups (N = 31; n = 8 young SAMR1, n = 8 aged SAMR1, n = 8 young SAMP8, n = 7 aged SAMP8). Kruskal-Wallis tests were performed to evaluate overall differences in the distributions of immune cell frequencies and ratios across study groups. Statistical significance was derived from two-sided tests and adjusted for multiple testing across all features using the Benjamini-Hochberg method (P_adj_). For significant results, leave-one-out sensitivity analyses were performed by recalculating Kruskal-Wallis tests iteratively after removing one sample at a time. The percentage of iterations for which the results remained significant are reported as “% sign.”. When the Kruskal-Wallis test was significant, post-hoc Dunn’s tests were conducted to assess pairwise differences between study groups. Effect sizes for Kruskal-Wallis tests were reported as ordinal epsilon squared (ε^2^_R_), calculated from the test-derived H-statistic divided by sample size minus one, and effect sizes for Dunn’s tests were reported as “r”, calculated as the test-derived Z-statistic divided by the square root of sample size. “N” indicates the total number of samples for the Kruskal-Wallis test. “N (pairwise)” indicates the combined sample size of the two groups being compared in each post hoc Dunn’s test.

**Supplementary Table S09.** Differences of T cell frequencies and ratios between study groups in female mice and male mice separately, and across all study groups including both sexes (n = 4 per sex, per genotype, per age, except the aged SAMP8 male group, n = 3). Only T cell frequencies and ratios that were identified as significant in the Kruskal-Wallis test performed across all samples (males and females combined; see Supplementary Table S08) were included in this sex-stratified analysis. Kruskal-Wallis tests were conducted to evaluate overall differences in the distributions of the selected T cell features across the four female study groups, the four male study groups, and all eight study groups combined. Statistical significance was assessed using two-sided tests with a significance threshold of α = 0.1, and p-values were adjusted for multiple testing across all features using the Benjamini-Hochberg method (P_adj_). When the Kruskal-Wallis test was significant, post-hoc Dunn’s tests were conducted to assess pairwise differences between study groups. Effect sizes for Dunn’s tests were reported as “r”, calculated as the test-derived Z-statistic divided by the square root of sample size. “N” indicates the total number of samples for the Kruskal-Wallis test. “N (pairwise)” indicates the combined sample size of the two groups being compared in each post hoc Dunn’s test. The first and second sections of this table present the female-only and male-only analyses, respectively, whereas the third section presents the combined eight-group analysis, including female-versus-male comparisons within genotype- and age-matched study groups.

**Supplementary Table S10.** RDA model association statistics calculated on the spleen T cell composition variation (Euclidean distance) in SAMR1 and SAMP8 mice (N = 31; n = 8 young SAMR1, n = 8 aged SAMR1, n = 8 young SAMP8, n = 7 aged SAMP8) using immune cell frequencies (scaled). Explanatory power and significance of all included variables are reported using single variable RDA and stepwise multivariable RDA. Explanatory power R^2^ and R^2^ adjusted (a modified version accounting for the number of predictors) are reported as “r2” and “r2adj”, respectively. P values are adjusted for multiple testing correction using the Benjamini-Hochberg method and are reported as “P_adj_”.

**Supplementary Table S11.** dbRDA model association statistics calculated on gut microbiome variation (genus-level Bray-Curtis dissimilarity) in SAMR1 and SAMP8 mice using the variables strain, age (weeks), age (category), sex and immune cell frequencies. Explanatory power and significance of all included variables are reported using single dbRDA and stepwise multivariable dbRDA. Explanatory power R^2^ and R^2^ adjusted (a modified version accounting for the number of predictors) are reported as “r2” and “r2adj”, respectively. P values are adjusted for multiple testing correction using the Benjamini-Hochberg method and are reported as “P_adj_”.

**Supplementary Table S12.** Association between specific microbiome features (Shannon diversity and specific relative genus abundances) and T cell frequencies as well as ratios in SAMR1 and SAMP8 mice (N = 31; n = 8 young SAMR1, n = 8 aged SAMR1, n = 8 young SAMP8, n = 7 aged SAMP8), calculated by Spearman test. Spearman’s rank correlation coefficient (rho) is used as the effect size. Statistical significance was derived from two-sided testing and adjusted for multiple testing correction using the Benjamini-Hochberg method. For significant results, leave-one-out sensitivity analyses were performed by recalculating Spearman tests iteratively after removing one sample at a time. The percentage of iterations for which the results remained significant are reported as “% sign.”.

**Supplementary Table S13.** Differences of MetaCyc pathway abundances between study groups (n = 8 per genotype, per age). Kruskal-Wallis tests were performed to evaluate overall differences in the distributions of pathway abundances across study groups. Statistical significance was assessed using two-sided tests with a significance threshold of α = 0.1, and p-values were adjusted for multiple testing across all features using the Benjamini-Hochberg method (P_adj_). When the Kruskal-Wallis test was significant, post-hoc Dunn’s tests were conducted to assess pairwise differences between study groups. Effect sizes for Kruskal-Wallis tests were reported as ordinal epsilon squared (ε^2^_R_), calculated from the test-derived H-statistic divided by sample size minus one, and effect sizes for Dunn’s tests were reported as “r”, calculated as the test-derived Z-statistic divided by the square root of sample size. “N” indicates the total number of samples for the Kruskal-Wallis test. N (pairwise) indicates the combined sample size of the two groups being compared in each post hoc Dunn’s test.

**Supplementary Table S14.** Association between specific MetaCyc pathway abundances and specific relative genus abundances (n = 8 per genotype, per age) as well as T cell frequencies and ratios (N = 31; n = 8 young SAMR1, n = 8 aged SAMR1, n = 8 young SAMP8, n = 7 aged SAMP8), calculated by Spearman test. Spearman’s rank correlation coefficient (rho) is used as the effect size. Statistical significance was derived from two-sided testing and adjusted for multiple testing correction using the Benjamini-Hochberg method.
